# Multi-Omics Analysis Unravels the Biosynthesis and Regulatory Mechanisms of Floral Scent Across Various Cultivars and Developmental Stages in *Phalaenopsis*

**DOI:** 10.3390/plants14233682

**Published:** 2025-12-03

**Authors:** Huaiqin Zhong, Yan Chen, Shengyuan Zhong, Jun He, Bing Lin, Jianshe Wu, Ronghui Fan

**Affiliations:** Institute of Crop Sciences, Fujian Academy of Agricultural Sciences (Fujian Germplasm Resources Center)/Fujian Engineering Research Center for Characteristic Floriculture, Fuzhou 350013, China

**Keywords:** *Phalaenopsis*, floral scent, transcriptional factor, terpenoid, ester compounds

## Abstract

*Phalaenopsis* is one of the most economically valuable genera in the Orchidaceae family. However, the common varieties of *Phalaenopsis* in the market rarely have fragrance, greatly limiting the sustainable development of the *Phalaenopsis* industry. Here, an integrated investigation was conducted on the patterns and determinants of aroma release in *Phalaenopsis*. GC-MS/MS analysis revealed that the primary volatile organic compounds (VOCs) in 10 fragrant *Phalaenopsis* cultivars are consistent. Terpenoids, alcohols, ketones, and esters collectively accounted for an average of 66.59% of the total VOCs across these 10 varieties. By performing metabolomic and transcriptomic analyses, we investigated the variation in 1532 VOCs in four different developmental stages of *Phalaenopsis* Formosa Sweet Memory. Metabolite analysis revealed that the levels of total volatiles, terpenoids, esters, and heterocyclic compounds were significantly upregulated during the flowering stages, and Linalool, β-Ocimene, and Methyl Benzoate were selected as key metabolites. While analyzing the correlation network between aroma components synthesis and differentially expressed genes, 33 key structural genes were detected and regulated by transcription factors. *PAXXG356500_TPS*, *PAXXG333030_4CL,* and *PAXXG061420_SAM* were key genes in the terpenoids and esters’ biosynthetic pathway, and they were co-expressed with aroma release. In summary, this study characterized the key metabolic pathways involved in aroma formation in *Phalaenopsis* and constructed the corresponding transcriptional regulatory network. These results laid a theoretical foundation for the subsequent research on aroma of *Phalaenopsis* and genetic engineering technology breeding.

## 1. Introduction

Floral scent serves as a vital criterion for evaluating plant quality. It also acts as an essential communication signal, enabling plants to exchange information with pollinators, defenders, and members of their own species [1]. The biosynthesis of floral scent compounds is a complex process within plant secondary metabolism, with volatile organic compounds (VOCs) as its products defining the unique aroma of flowers. According to their biosynthetic pathways, these compounds can be classified into terpenoids, phenylpropanoids/benzenoids, and fatty acids and their derivatives [2,3,4]. Terpenoids are a key part of the volatile compounds in floral scent, which are biosynthesized via two distinct pathways: the mevalonic acid (MVA) pathway and 2-C-methyl-D-erythritol-4-phosphate (MEP) pathway [5,6]. In the MEP pathway, pyruvate and glyceraldehyde 3-phosphate (G3P) serve as precursors that are transformed into isopentenyl diphosphate (IPP) or dimethylallyl diphosphate (DMAPP) through seven enzymatic steps [7,8,9]. Through catalysis by geranyl diphosphate synthase (GPPS) and terpene synthase (TPS), the precursors IPP and DMAPP give rise to monoterpene compounds, such as Linalool, β-Ocimene, and Pinene [7,8,9]. The aroma of orchids such as *Phalaenopsis* [10,11], *Oncidium* [12,13,14], and *Dendrobium* [15] is mainly composed of monoterpenes. In the phenylpropanoids/benzenoids pathway, phenylalanine is catalyzed by phenylalanine ammonia lyase (PAL) to form cinnamic acid [16]. After a series of enzymatic reactions, a wide variety of aromatic substances, such as Methyl Benzoate, are generated [16]. Within the genus *Dendrobium*, the volatile profiles of *Dendrobium lohohense* and *Dendrobium polyanthum* Wall. ex Lindl. were predominantly composed of esters, specifically methyl salicylate and octyl acetate, respectively [17]. At present, significant progress has been made in understanding the formation mechanism, regulatory network, and evolution and function of floral scent [18,19]. However, there is considerable variation in the composition and abundance of floral scent components among different species.

*Phalaenopsis* is one of the most economically valuable genera in the Orchidaceae family, known for its elegant flower pattern and long ornamental period [20,21]. Meanwhile, *Phalaenopsis* is the most industrialized variety in the Orchidaceae family, occupying a dominant position in the Asian, European, and North American markets [20,21]. During the long-term breeding process, the excessive pursuit of the quantity and size of flowers has resulted in *Phalaenopsis* cultivars lacking the floral fragrance trait [22,23]. Therefore, the common varieties of *Phalaenopsis* in the flower market rarely have fragrance. As its important biological characteristics and commercial properties, floral fragrance has gradually become a research hotspot in recent years. Due to the constraints of genetic background, genomic ploidy, and hybrid affinity, the breeding process of *Phalaenopsis* fragrant is slow [20,24]. Thus, understanding the molecular mechanism of aroma in *Phalaenopsis* is key to regulating the scent production process and improving breeding efficiency.

At present, there is limited research on the volatile components of *Phalaenopsis* flowers, primarily focused on a few native and commercial varieties. The main components of different species and varieties of *Phalaenopsis* are different, mainly terpene compounds. In native species, the strong-scented of *Phalaenopsis bellina* and *Phalaenopsis violacea* were determined by monoterpenes such as Linalool, Geraniol, and their derivatives [10,11]. The volatile components in the light-scented of *Phalaenopsis schilleriana* were mainly terpenoids and esters, while the non-scented of *Phalaenopsis equestris* was mainly composed of fatty acid derivatives [25,26]. Tong et al. [27] identified the aroma components of eight hybrid varieties of *Phalaenopsis*. Among them, the aroma of four varieties was mainly Linalool, and the aroma of three varieties was determined by α-Bergamotene. Xiao et al. [28] compared two scented types with two non-scented types of *Phalaenopsis* and found that the main fragrant components of *Phalaenopsis* flowers were Linalool and Geraniol. So far, the biosynthesis of the scent of *Phalaenopsis* is still unclear, with only a few research reports. Hsiao et al. [26] identified a group of floral scent production enzymes in the biosynthetic pathway from G3P to Geraniol, Linalool, and their derivatives of the strong-scented of *Phalaenopsis bellina* through the EST database (dbEST). In addition, the transcripts preferentially expressed in *Phalaenopsis bellina* were identified by comparison with non-scented of *Phalaenopsis equestris*, including transcripts encoding *lipoxygenase* (*LOX*), *epimerase*, *diacylglycerol kinase* (*DGK*), and *GPPS* [26]. Chuang et al. [28] identified the key transcription factor, *PbbHLH4*, regulating monoterpene synthesis by comparing transcriptome data of *Phalaenopsis bellina* and *Phalaenopsis aphrodite*. Instantly expressing *PbbHLH4* in non-scented *Phalaenopsis aphrodite* resulted in a 950-fold increase in monoterpene yield [28]. These studies provided a basis for investigating the regulatory mechanisms of scent production in *Phalaenopsis*. At present, research mainly focuses on a few varieties, making it difficult to explain the main characteristics and formation mechanisms of volatile components in *Phalaenopsis*.

In this study, we employed 10 fragrant *Phalaenopsis* cultivars to explore their key aroma compounds and further analyzed the aroma components during the flowering process of *P.* Formosa Sweet Memory (FSM). An integrated approach combining metabolomics, transcriptomics, and bioinformatics was employed to investigate the mechanisms underlying aroma formation in *Phalaenopsis*. The volatiles released by the *Phalaenopsis* were collected at different varieties and flowering stages using dynamic headspace technique and analyzed using the GC-MS/MS. Integrated with transcriptome sequencing data, this study identified and characterized key structural genes and transcription factors implicated in VOC biosynthesis in FSM. These findings establish a foundation for deciphering the aroma release mechanisms in *Phalaenopsis* and provide reference for genetic engineering breeding of floral aroma.

## 2. Results

### 2.1. Metabolite Analysis of VOCs Reveals the Variation in Floral Scent of 10 Phalaenopsis Cultivars

**Figure 1:**
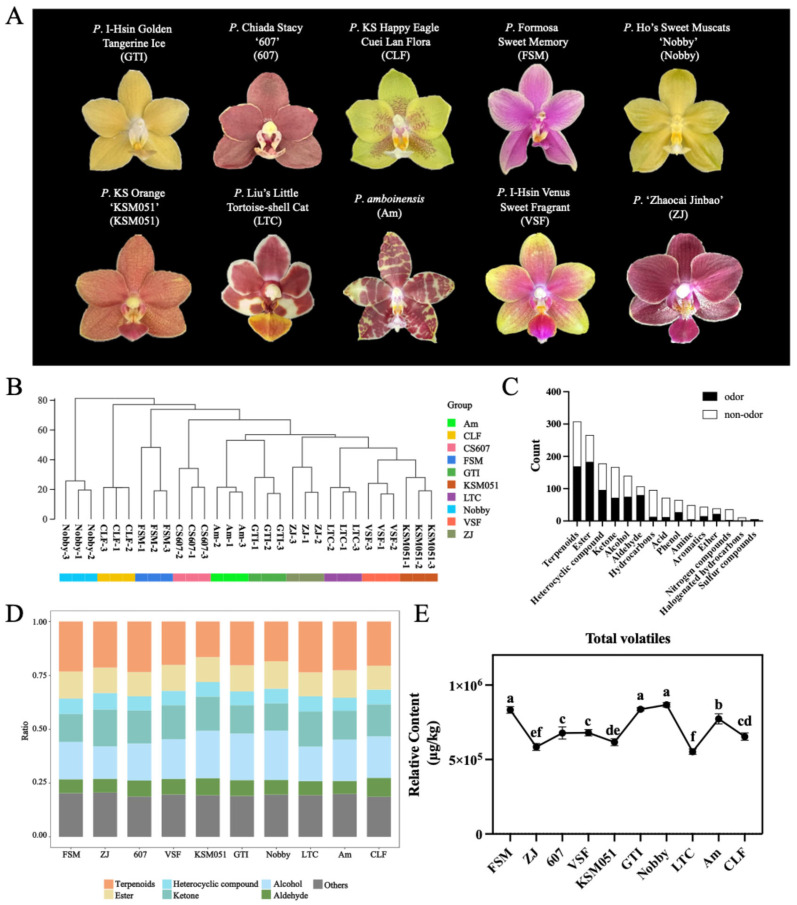
The metabolite profiling in the flowers of 10 fragrant *Phalaenopsis* cultivars. (**A**) Single flowers of 10 fragrant *Phalaenopsis* cultivars. (**B**) Cluster tree diagram of 10 samples. (**C**) The metabolite classes derived from 1583 VOCs based on their structures. (**D**) The proportion of metabolite categories derived from 1583 VOCs. The top six categories are displayed, while the remaining categories are classified as “other” categories. (**E**) Change trends of the contents of total VOCs of 10 fragrant *Phalaenopsis* cultivars. Values represent the mean ± SD (n = 3). Different lowercase letters indicate statistically significant differences (*p* < 0.05) among cultivars. Ten fragrant phalaenopsis cultivars were included: *Phalaenopsis* I-Hsin Golden Tangerine Ice (GTI), *Phalaenopsis* Chiada Stacy ‘607’ (607), *Phalaenopsis* KS Happy Eagle Cuei Lan Flora (CLF), *Phalaenopsis* Formosa Sweet Memory (FSM), *Phalaenopsis* Ho’s Sweet Muscats ‘Nobby’ (Nobby), *Phalaenopsis* KS Orange ‘KSM051’ (KSM051), *Phalaenopsis* Liu’s Little Tortoise-Shell Cat (LTC), *Phalaenopsis amboinensis* (AM), *Phalaenopsis* I-Hsin Venus Sweet Fragrant (VSF), and *Phalaenopsis* ‘Zhaocai Jinbao’ (ZJ).

In order to identify the volatile organic compounds (VOCs) related to the scent of *Phalaenopsis*, the Gas Chromatography–Tandem Mass Spectrometry (GC-MS/MS) method was used to analyze the volatile substances of 10 *Phalaenopsis* cultivars (Figure 1A). Assessment of the mixed quality control sample’s total ion flow diagram revealed highly reproducible qualitative results (Figure S1A). Furthermore, over 75% of the VOCs in the quality control samples exhibited a coefficient of variation (CV) below 0.3 (Figure S1B), indicating the reliability of the measurement results.

The metabolite profiling of VOCs revealed distinct varietal variations, as shown by cluster analysis and PCA (Figure 1B and Figure S1C). Six cultivars, namely *P. amboinensis* (Am), *P.* I-Hsin Golden Tangerine Ice (GTI), *P.* ‘Zhaocai Jinbao’ (ZJ), *P.* Liu’s Little Tortoise-shell Cat (LTC), *P.* I-Hsin Venus Sweet Fragrant (VSF), and *P.* KS Orange ‘KSM051’ (KSM051), formed a closely distributed cluster. The other four cultivars, *P.* Ho’s Sweet Muscats ‘Nobby’ (Nobby), *P.* KS Happy Eagle Cuei Lan Flora (CLF), *P.* Formosa Sweet Memory (FSM), and *P.* Chiada Stacy ‘607’ (607), were significantly separated (Figure 1B and Figure S1C). From the 10 cultivars, 1583 VOCs were detected and categorized into 16 metabolite categories (Figure 1C). The six most abundant metabolite categories were terpenoids, esters, heterocyclic compound, ketone, alcohol, and aldehydes. Among VOCs, terpenoid was the largest category (308 out of 1583), accounting for an average of 20.97% of the total intensity. The second largest category was ester compounds (266 out of 1583), accounting for an average of 16.80% of the total strength. The analysis of aroma metabolites was then performed according to their total content. Terpenoids, alcohols, ketones, and esters, identified as the top four VOC categories, represented an average of 66.59% of the total VOCs across all 10 cultivars (Figure 1D). The metabolite classification results indicated that the main VOCs were consistent among the 10 varieties. We observed the total VOCs content of 10 varieties and found that FSM, GTI, and Nobby were significantly higher than others (Figure 1E). It can be inferred that FSM, GTI, and Nobby have a stronger ability to regulate aroma synthesis at the transcriptional level and were good materials for studying the molecular mechanism of floral fragrance synthesis.

### 2.2. Identification of VOCs Related Regulatory Factors During Different Floret Stages of Phalaenopsis Formosa Sweet Memory

**Figure 2:**
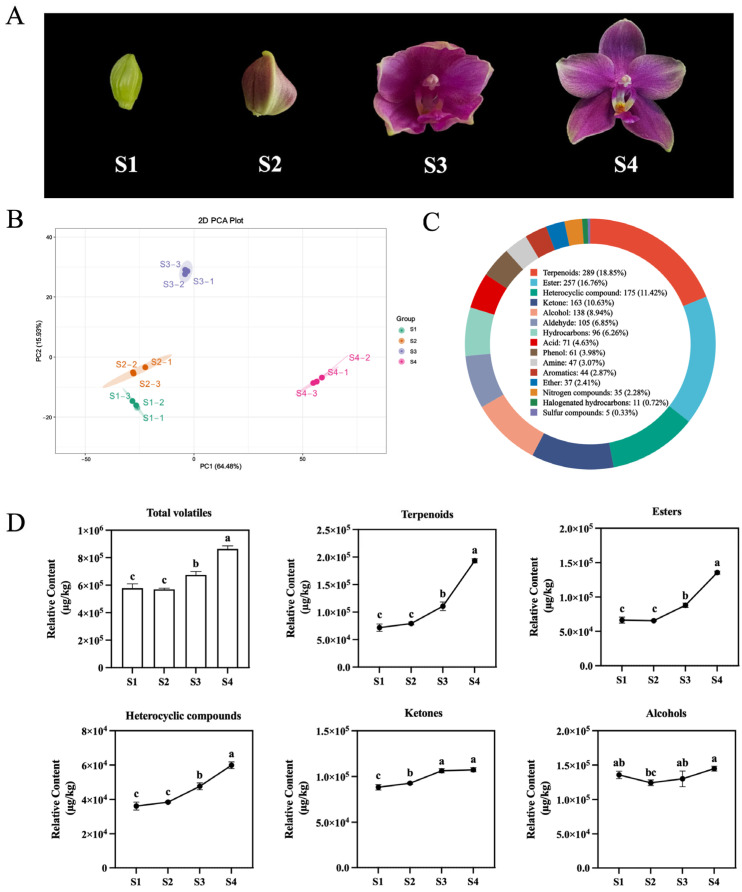
Metabolite analysis for different floret stages of *Phalaenopsis* Formosa Sweet Memory. (**A**) The four different opening stages of the FSM. (**B**) Principal component analysis (PCA) on 1532 VOCs in FSM during different flowering stages. (**C**) Classification and proportion of VOCs detected in different flowering stages (S1, S2, S3, and S4) of the FSM. (**D**) Change trends of the contents of total VOCs, terpenoids, esters, heterocyclic compounds, ketones, and alcohols during different FSM flowering stages. Values represent the mean ± SD (n = 3). Different lowercase letters indicate statistically significant differences (*p* < 0.05) among stages.

To further identify the VOCs associated with *Phalaenopsis* aroma, the volatile substances of FSM at different flowering stages were analyzed (Figure 2A). The application of principal component analysis (PCA) and Pearson’s correlation coefficient indicated a clear distinction among samples, corresponding to different floret stages (Figure 2B and  Figure S2A). The results also showed primary clustering in accordance with the three biological replicates. In addition, the total ion flow diagram of the mixed quality control sample and CV indicated the reliability of the measurement results (Figure S2B,C). Among the 16 volatile organic compounds, terpenoids (18.85%), ester (16.76%), heterocyclic compound (11.42%), ketone (10.63%), and alcohol (8.94%) rank as the top five in terms of relative content (Figure 2C). We calculated the content of total volatiles and the top five chemical groups, and we found that they were synthesized in large quantities at the flower-opening stage (S4) (Figure 2D). The total volatiles were significantly upregulated during the opening stages of *Phalaenopsis*, while terpenoids, esters, and heterocyclic compounds showed the same trend (Figure 2D). Consequently, it was reasonable to speculate that the half-flowering stage (S3) and flower-opening stage (S4) were the key stages for the formation and release of the aroma of *Phalaenopsis* Formosa Sweet Memory.

**Figure 3:**
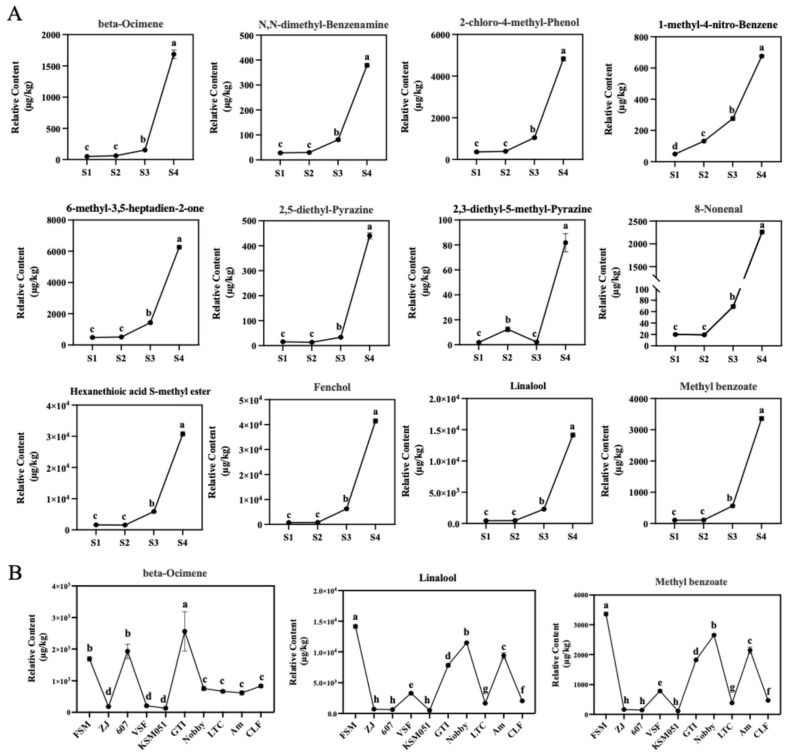
Contents of volatile organic compounds (VOCs) in *Phalaenopsis* flowers at different stages. (**A**) Change trends of the contents of key odorants at different FSM flowering stages. Significantly differential VOCs between intergroups accorded with OAVs > 1 and log2 fold change (FC) ≥ 1. (**B**) Change trends of the contents of β-Ocimene, Linalool, and Methyl Benzoate of 10 fragrant *Phalaenopsis* cultivars. Values represent the mean ± SD (n = 3). Different lowercase letters indicate statistically significant differences (*p* < 0.05).

To further explore the key odorants that form the aroma of *Phalaenopsis*, we applied the K-means clustering algorithm to classify the distinct profiles of differential metabolites. Seven different expression classes (class 1 to class 7) were directly presented, with 27, 36, 172, 123, 125, 398, and 80 members, respectively (Figure S3A). Based on the changes in the total volatiles, we found that the trends of class 5 and class 6 were similar, containing a total of 523 members (Figure S3). We also calculated the relative odor activity values (rOAVs) for identified VOCs, 12 of which had OAVs > 1 at four stages, namely β-Ocimene, N,N-dimethyl-Benzenamine, 2-chloro-4-methyl-Phenol, 1-methyl-4-nitro-Benzene, 6-methyl-(E)-3,5-Heptadien-2-one, 2,5-diethyl-Pyrazine, 2,3-diethyl-5-methyl-Pyrazine, 8-Nonenal, Hexanethioic acid S-methyl ester, Fenchol, Linalool, and Methyl Benzoate (Figure 3A). Among them, Linalool, β-Ocimene, and Methyl Benzoate are important components of floral scent in Orchidaceous plants. It is worth noting that the content trends of Linalool and Methyl Benzoate in the 10 *Phalaenopsis* cultivars were similar to their total VOC content, while FSM, GTI, Nobby, and Am were significantly higher than those of other cultivars (Figure 3B). Meanwhile, high levels of β-Ocimene were also detected in FSM and GTI (Figure 3B). Thus, these traits served as phenotypic data for investigating the underlying regulatory mechanisms of *Phalaenopsis* aroma formation.

### 2.3. Expression of 82 TF Genes During Phalaenopsis Opening Was Positively Correlated with Aroma-Related Structural Genes

**Figure 4:**
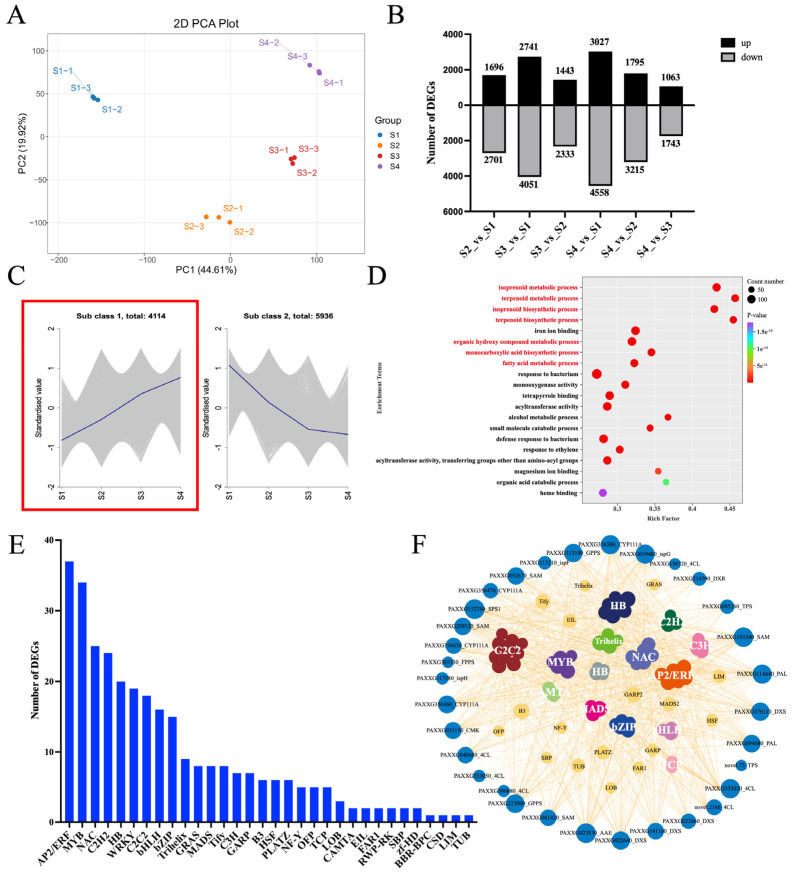
Expression patterns of differentially expressed genes (DEGs) in *Phalaenopsis* at different flowering stages. (**A**) PCA on FPKM in *Phalaenopsis* at different flowering stages. (**B**) Differentially expressed genes (DEGs) at different flowering stages. Black bars: upregulation; gray bars: downregulation. (**C**) K-means clustering reveals two major expression trends of DEGs during flowering. The gray background displays the result of adding or subtracting the standard deviation from the average value. The lines represent the average values. The expression of genes in the red box shows a similar pattern to the aroma content during *Phalaenopsis* flowering. (**D**) KEGG pathway enrichment analysis of DEGs in Cluster 1. The red font indicates the GO enrichment pathways related to aroma synthesis. (**E**) Classification of transcription factor (TF) families in Cluster 1. (**F**) Co-expression network of TFs and aroma-related genes in Cluster 1 was drawn by Cytoscape (v. 3.5.1) software based on the expression correlation (Pearson’s correlation coefficient ≥ 0.9, *p* ≤ 0.01). The outer blue spheres represent the structural genes related to the aroma biosynthesis pathway, while the differently colored spheres inside represent different transcription factor families.

To further elucidate the molecular regulatory mechanism of aroma formation, we conducted RNA-seq analysis of the flowering process of *Phalaenopsis* Formosa Sweet Memory. The PCA indicated a clear distinction among samples from different flowering stages, mainly based on the repeated clustering of three biological replicates (Figure 4A). We analyzed the differentially expressed genes (DEGs) for S2 vs. S1 (4397), S3 vs. S1 (6792), S3 vs. S2 (3776), S4 vs. S1 (7585), S4 vs. S2 (5010), and S4 vs. S3 (2806) combinations, generating a total of 10,050 DEGs (Figure 4B). In order to further explore the biosynthesis of VOCs during the flowering process, the K-means clustering algorithm was used to classify the different characteristics of the DEG expression profile. This analysis revealed two different expression clusters (Cluster 1 and Cluster 2), comprising 4114 and 5936 genes, respectively (Figure 4C). Based on the changes in the content of key odorants and gene expression profiles, we found that the gene expression in Cluster 1 exhibited a similar upregulation pattern to the increasing aroma content during *Phalaenopsis* flowering (Figure 4C). Therefore, we performed a functional analysis of Cluster 1. The GO enrichment analysis demonstrated that these genes were significantly involved in the “isoprenoid metabolic process”, “terpenoid metabolic process”, “isoprenoid biosynthetic process”, “terpenoid biosynthetic process”, “organic hydroxy compound metabolic process”, “monocarboxylic acid biosynthetic process”, and “fatty acid metabolic process” (Figure 4D). Based on the Linalool, β-Ocimene, and Methyl Benzoate biosynthetic pathways, 47 structural genes involved in the aroma formation of *Phalaenopsis* were identified (Figure S4). These genes may collectively contribute to aroma formation in *Phalaenopsis*.

Transcription factors (TFs) can specifically bind to specific DNA sequences of genes, and they are core components of the gene transcriptional regulatory network. From Cluster 1, we identified 307 TFs, distributed across 32 families (Figure 4E). Among these TF genes, 82 genes from 30 TF families were highly positively correlated with VOCs during the development of *Phalaenopsis*. A total of 1883 TF-structural genes showed a highly significant positive correlation (r ≥ 0.9), such as *C2C2*, *HB*, *MYB*, *AP2*/*ERF*, *bHLH*, *NAC*, *C3H*, *TCP*, *CAMTA*, *MADS*, *Trihelix*, and *bZIP* (Figure 4F). This suggests that these TFs may indirectly regulate aroma formation by modulating the expression of aroma-related structural genes.

### 2.4. Thirty-Three Structural Genes and TF Genes Were Hub Genes Involved in the Aroma Synthesis During Phalaenopsis Flowering

**Figure 5:**
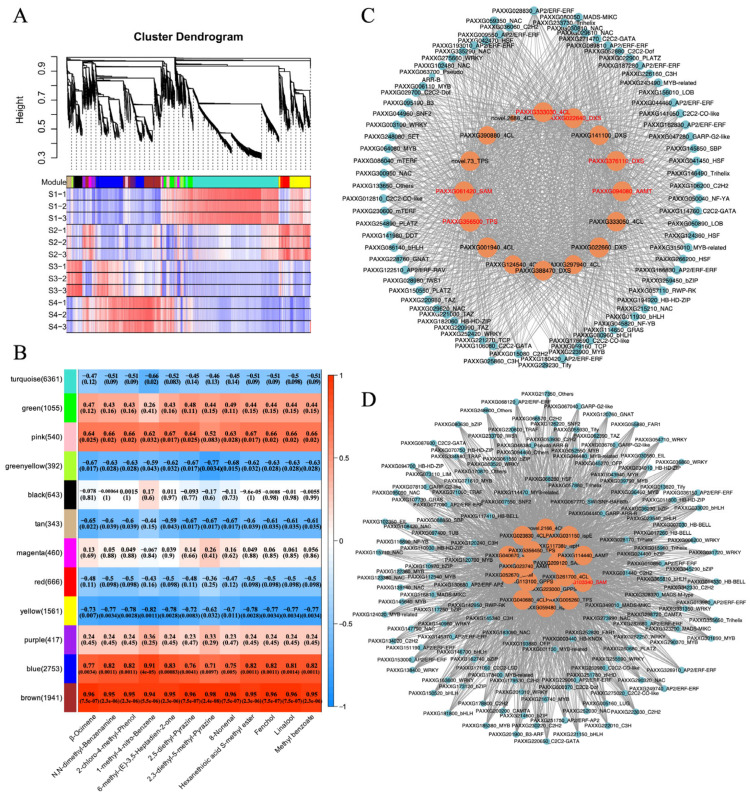
Weighted gene co-expression network analysis (WGCNA) of differentially expressed genes (DEGs) during *Phalaenopsis* flowering. (**A**) Hierarchical clustering tree of the co-expression modules. The major tree branches constitute 12 distinct co-expression modules. The bottom part shows the clustering heatmap of genes within the module, with red indicating high expression, and purple indicating low expression. (**B**) The correlation relationships between the modules were analyzed through the Pearson’s correlation coefficients. The numbers on the left side of the modules represent the number of genes in each module, and the numbers in the heatmap represent the *p*-values and correlation coefficients, respectively. (**C**) The co-expression network of structural genes related to the synthesis of terpenoids and esters in the MEbrown. The expression levels of red font genes were positively correlated with the contents of terpenoids and esters in different flowering stages of *Phalaenopsis* (*p*-value < 0.01, correlation coefficient ≥ 0.9), and they were differentially expressed in S2 vs. S3 and S3 vs. S4. (**D**) The co-expression network of structural genes related to the synthesis of terpenoids and esters in the MEblue. The expression levels of red font genes were positively correlated with the contents of terpenoids and esters in different flowering stages of *Phalaenopsis* (*p*-value < 0.01, correlation coefficient ≥ 0.9), and they were differentially expressed in S2 vs. S3 and S3 vs. S4.

To explore the gene regulatory network underlying aroma formation during the *Phalaenopsis* flowering, we performed a Weighted Gene Co-Expression Network Analysis (WGCNA) to identify co-expressed gene modules. A total of twelve modules (labeled turquoise, green, pink, green-yellow, black, tan, magenta, red, yellow, purple, blue, and brown) were classified, with 6361, 1055, 540, 392, 643, 343, 460, 666, 1561, 417, 2753, and 1941 members, respectively (Figure 5A,B). The genes within the same module exhibited a high degree of correlation and co-expression trends (Figure S5). To further investigate the hub genes associated with aroma formation during *Phalaenopsis* flowering, the content of key odor compounds in the corresponding samples was utilized as phenotypic data to analyze gene–module–trait correlations. This analysis revealed that the MEbrown was highly positively correlated with 12 key odor compounds, and the MEblue was highly positively correlated with 8 key odor compounds (correlation coefficient ≥ 0.8) (Figure 5B). The expression of MEbrown and MEblue genes was significantly upregulated and reached its peak in the flower-opening stage (S4), consistent with the trend of changes in aroma content (Figure S5). Subsequently, we conducted KEGG enrichment analysis on the genes in the MEblue and MEbrown (Figure S6). The results indicated that the genes of MEblue were mainly enriched in “Biosynthesis of secondary metabolites”, “Glycerophospholipid metabolism”, “alpha-Linolenic acid metabolism”, “Phenylalanine, tyrosine and tryptophan biosynthesis”, “Fatty acid degradation”, and “Terpenoid backbone biosynthesis” (Figure S6A). The genes in MEbrown were mainly enriched in “Biosynthesis of secondary metabolites”, “Biosynthesis of various plant secondary metabolites”, “Glyoxylate and dicarboxylate metabolism”, and “Pentose phosphate pathway” (Figure S6B). These pathways are related to the biosynthesis of key odor substances.

To identify TFs highly connected to the aroma synthesis in *Phalaenopsis*, we exported the selected modules (MEbrown and MEblue) and visualized them using Cytoscape (Figure 5C, D). The 16 structural genes in the MEbrown were regulated by 44 TFs, including *4CL* (PAXXG001940, PAXXG124540, PAXXG297940, PAXXG333030, PAXXG333050, PAXXG390880, and novel.2686), *DXS* (PAXXG022640, PAXXG022660, PAXXG141100, PAXXG376110, and PAXXG388470), *TPS* (PAXXG356500 and novel.73), *SAM* (PAXXG061420), and *AAMT* (PAXXG094080) (Figure 5C). These TFs belong to 25 families, such as *AP2*/*ERF* (10), *NAC* (8), *C2C2* (8), *MYB* (5), and *HSF* (4). Within the MEblue module, we identified 146 TFs from 39 families, the most prominent being the MYB family (including MYB-related), with 18 members (Figure 5D). These results indicated that these TFs may regulate aroma formation in *Phalaenopsis* through the TF-structural gene regulatory network. We conducted a Pearson’s correlation coefficient analysis, comparing these TFs with the relevant key odor substances. The expression levels of these genes (FPKM > 10) exhibited strong positive correlations with the abundance of key odorants that peaked at the S4 stage (*p*-value < 0.01, correlation coefficient ≥ 0.9) (Figure S6C). These TFs may be related to the odor formation of *Phalaenopsis*. In the regulatory network of the MEbrown and MEblue, we further screened structural genes related to the synthesis of terpenoids and esters. A total of seven aroma-related structural genes were screened, including *DXS* (PAXXG022640 and PAXXG376110) and *TPS* (PAXXG356500) related to terpenoids synthesis, and *4CL* (PAXXG333030), *SAM* (PAXXG061420 and PAXXG103340), and *AAMT* (PAXXG094080) related to esters synthesis (Figure 5C,D). The expression levels of these genes were positively correlated with the contents of terpenoids and esters in different flowering stages of *Phalaenopsis* (*p*-value < 0.01, correlation coefficient ≥ 0.9), and they were differentially expressed in S2 vs. S3 and S3 vs. S4 (Figure S6D). These findings implicated these genes in the aroma formation of *Phalaenopsis*.

### 2.5. Expression Patterns of Candidate DEGs Involved in Phalaenopsis Aroma Biosynthesis

**Figure 6:**
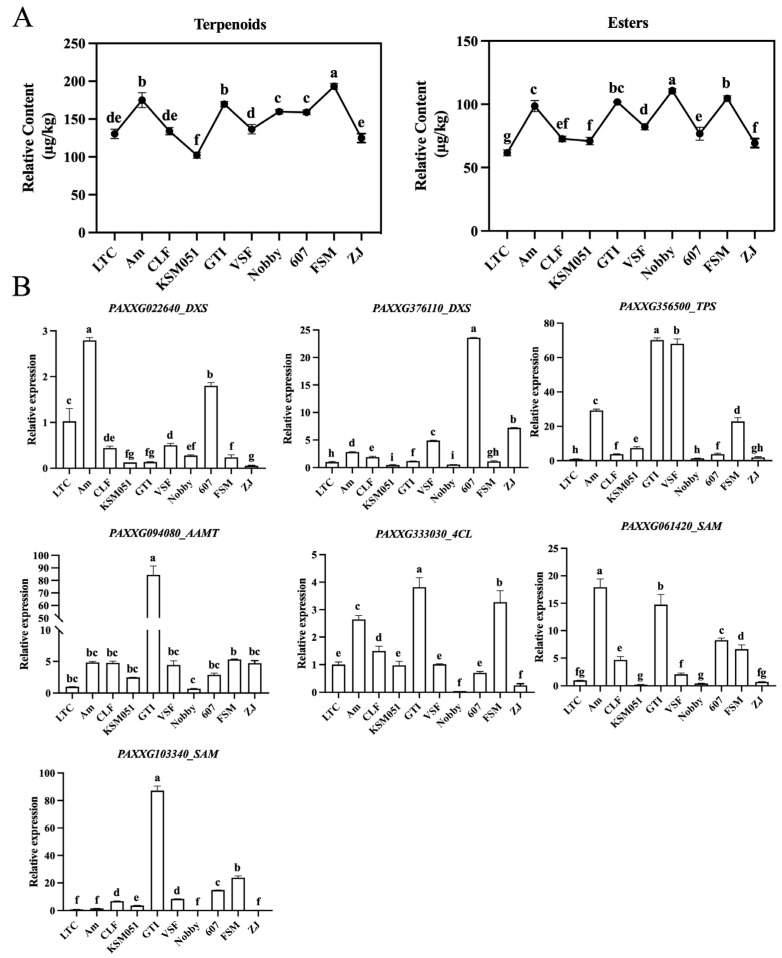
Integrated analysis of floral scent emission and biosynthetic genes expression of 10 fragrant *Phalaenopsis* cultivars. (**A**) Change trends of the contents of terpenoids and esters of 10 fragrant *Phalaenopsis* cultivars. (**B**) Relative expression of structural genes *PAXXG022640_DXS*, *PAXXG376110_DXS*, *PAXXG356500_TPS*, *PAXXG094080_AAMT*, *PAXXG333030_4CL*, *PAXXG061420_SAM*, and *PAXXG103340_SAM* of 10 fragrant *Phalaenopsis* cultivars. All treatments were performed with three biological replicates, each measured in triplicate. Data are presented as mean ± SD. Different lowercase letters denote statistically significant differences (*p* < 0.05).

To investigate the relationship between aroma synthesis genes and aroma release, we first quantified the content of VOCs released by esters and terpenoids in different varieties of *Phalaenopsis* (Figure 6A). The GC-MS/MS data indicated that the content of terpenoids in FSM, GTI, and Am were significantly higher than that in other varieties. Similar to total volatiles, esters were the highest in the three varieties of FSM, GTI, and Nobby. It was inferred that there were differences in aroma types among different varieties.

To assess the correspondence between the transcription of aroma-related structural genes and VOCs emission patterns, we quantified the expression of seven candidate structural genes (*PAXXG022640_DXS*, *PAXXG376110*_*DXS*, *PAXXG356500*_*TPS*, *PAXXG333030*_*4CL*, *PAXXG061420*_*SAM*, *PAXXG103340*_*SAM*, and *PAXXG094080*_*AAMT*) across multiple *Phalaenopsis* varieties using quantitative real-time PCR (qRT-PCR). Two *DXS* genes related to terpene synthesis were highly expressed in Am (*PAXXG022640_DXS*) and 607 (*PAXXG376110*_*DXS*), respectively. Notably, *PAXXG356500_TPS* was highly expressed in GTI, VSF, Am, and FSM, similar to the trend of terpenoids content changes among different varieties. The three structural genes (*PAXXG094080_AAMT*, *PAXXG333030_4CL*, and *PAXXG103340_SAM*) related to esters synthesis were highly expressed in GTI, while *PAXXG061420_SAM* was highly expressed in Am. Interestingly, both *PAXXG333030_4CL* and *PAXXG061420_SAM* were highly expressed in FSM, GTI, and Am, showing a similar trend to the changes in esters content among different varieties. It can be inferred that the synthesis of terpenoids and esters among different varieties of *Phalaenopsis* may be regulated by different genes, and *PAXXG356500_TPS, PAXXG333030_4CL*, and *PAXXG061420_SAM* played an important role in this process.

## 3. Discussion

### 3.1. The Main VOCs of Phalaenopsis Formosa Sweet Memory Were β-Ocimene, Linalool, and Methyl Benzoate

Flower scent, as a key signaling trait released by ornamental plants, also constitutes one of the core elements of their ornamental and economic value. Due to the common issues of weak fragrance, small flower quantity, small flower diameter, and poor fertility in *Phalaenopsis* aroma varieties, the commonly available varieties in the flower market rarely have fragrance [29]. Due to the genetic background, genome ploidy, and hybridization affinity limitations of *Phalaenopsis*, little is known about the synthesis and regulation pathways of its floral fragrance [20,24]. At present, various floral aroma components of *Phalaenopsis* have been identified. In the strong-scented type of *Phalaenopsis bellina*, monoterpenes, phenylpropanoids, benzenoids, and fatty acid derivatives were detected [26]. Among them, monoterpenoid compounds such as Geraniol, Linalool, and their derivatives accounted for more than 80% of all volatile substances [26]. The volatile components of the light-scented *Phalaenopsis schilleriana* mainly consist of terpenoids and esters, including neryl acetate, nerol, citronellol, and citronellyl acetate [25]. The primary volatile compounds detected in the scentless *Phalaenopsis equestris* were fatty acid derivatives, phenylpropanoids, and benzenoids, with no monoterpene derivatives detected [26]. Xiao et al. [11] found that monoterpenes and sesquiterpenes were the main components in *Phalaenopsis violacea*. By comparing the odorless type with the fragrant type of *Phalaenopsis*, they identified Linalool and Geraniol as the main odor causing compounds [30]. Based on our analysis, a total of 1583 VOCs were identified across 10 fragrant *Phalaenopsis* cultivars, with terpenoids and esters being the two most abundant categories. From this, it can be seen that terpenoids and esters are important components of the fragrance of *Phalaenopsis*. These 10 *Phalaenopsis* cultivars exhibit two patterns in the relative content of VOCs. Among them, six varieties (Am, GTI, ZJ, LTC, VSF, and KSM051) were closely distributed, while the other four varieties (Nobby, CLF, FSM, and 607) had significant differences. The diversity of volatile organic compounds across different plant cultivars is a common phenomenon, which provides critical guidance for the genetic improvement and molecular breeding of *Phalaenopsis* [31,32].

Terpenoids, particularly monoterpenoids, serve as the core chemical constituents responsible for the characteristic floral scent of *Phalaenopsis* and other flowering plants [26,33]. We have identified Linalool and β-Ocimene as the main volatile components of the FSM. Linalool, as a monoterpenoid alcohol, is widely present in plants and is known for its sweet, floral, and woody scents. It is considered as the core aroma component of orchid ornamental plants such as *Dendrobium* [34] and *Oncidium hybridum* [12,13]. β-Ocimene is a common acyclic monoterpene and one of the main components of many plant floral volatiles, such as *Lilium* [35], *Hedychium coronarium* [36], and *Narcissus tazetta* [37]. Linalool and β-Ocimene also collaborate to form the signature scent of *Phalaenopsis bellina* [26]. In addition to terpenoids, Methyl Benzoate (easter) has also been identified as a critical aroma compound in FSM. Methyl Benzoate is an important aromatic ester compound, which was widely present in plant volatiles and was an important component of the aroma of jasmine [38] and lily [39]. Meanwhile, the content trends of Linalool, β-Ocimene, and Methyl Benzoate in 10 varieties of *Phalaenopsis* were similar to their total volatile organic compound content. In previous studies, Linalool, β-Ocimene, and Methyl Benzoate have been proven to be important components of the aroma of Orchidaceae plants [40,41], among which Linalool is the key aroma of *Phalaenopsis* [42]. The unique aroma of the *Phalaenopsis* may be characterized by the combination of Linalool, β-Ocimene, and Methyl Benzoate.

### 3.2. PeTPS, Pe4CL, and PeSAM May Function as Master Regulators in the Coordinated Regulation of Phalaenopsis Aroma Formation

Floral fragrance is the result of the combined action of multiple volatile components, and the type and content of volatile components determine the floral aroma. The biosynthesis of specific metabolites is finely regulated at spatiotemporal levels through transcriptional control of structural genes associated with these pathways [43]. In this study, we found that the contents of monoterpenes (β-Ocimene and Linalool) and ester (Methyl Benzoate) significantly increased during the flowering process, and the structural genes related to these pathways were also significantly upregulated. In *Phalaenopsis*, we identified *DXS* (5), *TPS* (4), *ispE* (1), *ispH* (1), *ispG* (1), and *GPPS* (2) genes in the terpenoid synthesis pathway, which showed similar trends to the release of terpenoids. Meanwhile, 19 genes with similar esters release trends were identified, including *4CL* (12), *SAM* (4), and *AAMT* (3) genes. β-Ocimene and Linalool were monoterpenoids primarily synthesized via the MEP pathway. To identify the key genes regulating β-Ocimene and Linalool biosynthesis, this study analyzed DEGs associated with the MEP pathway. The MEP pathway generates IPP and DMAPP, which serve as the precursors for the biosynthesis of diverse monoterpenes by GPPS and TPS, leading to the production of various monoterpene compounds [44]. TPS is a key enzyme in the biosynthetic pathway of monoterpenoids. Consequently, studies on floral fragrance have predominantly centered on the identification and functional characterization of *TPS* genes [44]. The main aroma compounds of *Osmanthus* were β-Ocimene, Linalool, and Linalool derivatives, and three *TPS* genes have been identified to regulate the production of β-Ocimene or Linalool. Among them, *OfTPS1* and *OfTPS2* can promote the production of β-Linalool, while *OfTPS3* can regulate the content of trans-β-ocimene [45]. In *Lilium* ‘Siberia’, two terpene synthase genes (*LoTPS1* and *LoTPS3*) were highly expressed in sepals and petals, with *LoTPS1* and *LoTPS3* being responsible for the formation of (±)-Linalool and β-Ocimene [46]. Huang et al. [47] confirmed that both the *TPS-b* and *TPS-e/f* enzymes coordinated the biosynthesis of the floral monoterpene compounds in *Phalaenopsis bellina*. Correlation analysis revealed that the expression patterns of *PAXXG356500_TPS* in different varieties and at different stages of *Phalaenopsis* were positively correlated with β-Ocimene or Linalool. From this, it can be inferred that *PAXXG356500_TPS* was involved in regulating the synthesis of terpenoid compounds.

In plants, Methyl Benzoate is primarily synthesized via the phenylpropanoid pathway. The precursor benzoic acid, under the catalytic action of benzoic acid carboxyl methyltransferase, combines with a methyl group donated by S-adenosylmethionine (SAM) to ultimately form Methyl Benzoate through esterification [16]. 4-Coumaroyl-CoA Ligase (4CL) is a core rate-limiting enzyme in the phenylpropanoid pathway, acting as one of the “master switches” in the floral scent synthesis network. In *Osmanthus*, the *4CL* genes were specifically expressed in floral tissues, with very low expression levels in vegetative organs such as roots and leaves [48]. QRT-PCR analysis of six 4CL genes in *Rosa multiflora* revealed that most family members were predominantly expressed in flowers and young thorns [49]. In the core pathway of floral volatile compound biosynthesis, phenylpropanoid derivatives were major contributors to the floral scent of many plants [39,40]. During the synthesis of these compounds, several key steps rely on methylation reactions catalyzed by methyltransferases, and SAM serves as the exclusive methyl donor for these methyltransferases [16]. We identified *PAXXG333030_4CL* and *PAXXG061420_SAM*, whose expression pattern in *Phalaenopsis* correlated with the metabolic concentration changes in Methyl Benzoate, making it a candidate for further investigation.

### 3.3. The Formation of the Fragrance of Phalaenopsis May Be Regulated by Multiple Transcription Factors

The synthesis and accumulation of floral scent compounds constitute a complex process governed by the coordinated regulation of multiple genes. In this process, transcription factors function as core regulatory nodes that precisely control floral scent metabolism by binding to the promoter regions of downstream structural genes [19]. In this study, integrated volatilomics and transcriptomics analysis revealed that the expression of numerous TFs was significantly correlated with the emission of key scent compounds in *Phalaenopsis*. This included several TF families, such as C2C2, HB, MYB, AP2/ERF, bHLH, NAC, C3H, TCP, CAMTA, MADS, Trihelix, and bZIP. A large number of TFs have been reported to regulate the formation of aromatic compounds by controlling the expression of key genes in the aroma biosynthetic pathways. In *Cymbidium tracyanum*, transcription factors CtAP2/ERF1, CtAP2/ERF4, CtbZIP1, CtMYB2, and CtMYB3 can participate in the formation of terpenoids by activating the expression of *CtTPS* [50]. In *Lilium* ‘Siberia’, LiMYB1, LiMYB305, and LiMYB330 can directly bind to the promoter of the terpene synthase gene *LiTPS2*, thereby positively regulating the synthesis of the major monoterpene floral scent compounds [51]. In petunias, PhbZIP3 can bind to the *PhPAL2* and *PhBSMT* promoter regions to participate in the regulation of benzene/phenylpropane compound synthesis [52]. Xi et al. [53] identified that WRKY33 in osmanthus can synergistically induce the expression of *OfTPS7* and *OfDXS1*, thereby promoting the production of menthol. Based on these findings, we propose that these TFs form a regulatory network that underlies the synergistic biosynthesis of key aroma compounds in *Phalaenopsis*.

## 4. Materials and Methods

### 4.1. Plant Materials

The materials used in the current study were planted in the Characteristic Orchid Germplasm Resources Preservation Nursery (Fuzhou, China). Ten *Phalaenopsis* cultivars were examined: *P.* I-Hsin Golden Tangerine Ice (GTI), *P.* Chiada Stacy ‘607’ (607), *P.* KS Happy Eagle Cuei Lan Flora (CLF), *P.* Formosa Sweet Memory (FSM), *P.* Ho’s Sweet Muscats ‘Nobby’ (Nobby), *P.* KS Orange ‘KSM051’ (KSM051), *P.* Liu’s Little Tortoise-shell Cat (LTC), *P. amboinensis* (Am), *P.* I-Hsin Venus Sweet Fragrant (VSF), and *P.* ‘Zhaocai Jinbao’ (ZJ). In addition, the florets of FSM were sampled at four different flowering stages: S1 (early-stage bud), S2 (late-stage bud), S3 (half-flowering stage), and S4 (flower-opening stage). All fresh samples were immediately frozen at −80 °C after collection and maintained under these conditions until analysis, with three biological replicates per sample.

### 4.2. Collection and GC-MS/MS Analysis of VOCs

All samples were cryogenically ground using liquid nitrogen. Exactly 500 mg of the resulting powder was transferred to a headspace vial, followed by the addition of saturated NaCl solution and 20 μL of internal standard solution (10 μg/mL, 3-Hexanone-2,2,4,4-d4). The VOCs were extracted using headspace solid-phase microextraction (HS-SPME). Detailed instrument models and analytical parameters are provided in Table S1. The GC oven temperature program was set as follows: hold at 40 °C for 3.5 min; increase to 100 °C at 10 °C/min; raise to 180 °C at 7 °C/min; and then ramp to 280 °C at 25 °C/min, with a final hold time of 5 min. MassHunter software (v. B.08.00) was employed to process the mass spectrometry data. Compound identification was performed by comparing spectra against the Metare’s metabolite database (MWDB). Quantitative ions were selected for chromatographic peak integration and correction, and data normalization was carried out using the internal standard (3-Hexanone-2,2,4,4-d4, CAS: 24588-54-3) to ensure quantification accuracy.

### 4.3. RNA Extraction and Transcriptome Sequencing

Transcriptome sequencing samples were collected at four distinct developmental stages of flowering: S1 (early-stage bud), S2 (late-stage bud), S3 (half-flowering stage), and S4 (flower-opening stage). Total RNA was isolated from collected tissues by ethanol precipitation and CTAB-PBIOZOL. The integrity and concentration of the RNA were assessed using the Qsep400 high-throughput biological fragment analyzer and the Qubit fluorescence quantitative analyzer, respectively. High-quality mRNA was isolated using Oligo (dT) beads and then cleaved into small fragments. The cDNA was then purified, and the library was prepared through end repair, polyA tailing, and adapter ligation. DNA Nanoball Sequencing technology was used to sequence 12 libraries. The clean reads were mapped to the *Phalaenopsis aphrodite* reference genome utilizing HISAT2 [20,54].

### 4.4. Analysis of RNA-Seq Data

Gene expression levels were quantified using fragments per kilobase of transcript per million mapped reads (FPKM) as the standardized metric. Differential expression analysis was performed between sample groups using DESeq2 [55,56], with |log2Fold Change| ≥ 1 and FDR < 0.05 (Table S2). Principal component analysis (PCA) was conducted using FPKM values to determine the clustering situation and distribution pattern of the samples. Both PCA and correlation analysis were completed using the R version 3.5.1. The K-means cluster analysis for DEGs was performed using R version 4.2.0 [57], and KEGG was performed using the Metware Cloud (https://cloud.metware.cn, accessed on 7 July 2025). The TFs were predicted using iTAK software (v. 1.7a) [58], which integrates the PlnTFDB and PlantTFDB databases. Subsequently, a gene co-expression network was constructed using the WGCNA software package (v. 1.71) from the identified differentially expressed genes (DEGs) and transcription factors, and visualized using Cytoscape (v. 3.5.1). Based on the contents of 12 metabolic substances, a heatmap of the relationships between WGCNA modules and traits was plotted using R package (v. 4.2.2).

### 4.5. qRT-PCR Assay

cDNA synthesis was performed according to the instructions of PrimeScriptTM II 1st Strand cDNA Synthesis Kit (Takara Bio, Kyoto, Japan). RT-qPCR analysis was conducted by a QuantStudio 1 Plus system with ChamQ Universal SYBR qPCR Master Mix (Vazyme Biotech Co., Ltd., Nanjing, China). Relative expression was calculated using the 2^−ΔΔCT^ method, and the expression data were normalized using Actin as a reference gene. All primer sequences used in this study are listed in Table S3.

### 4.6. Statistical Analysis

The data were analyzed using one-way analysis of variance (ANOVA), and Duncan’s pairwise comparison test was employed to compare the means. In all figures, values labeled with different letters denote statistically significant differences at *p* < 0.05. The graphs were generated using GraphPad software (v. 10.5.0). 

## 5. Conclusions

**Figure 7:**
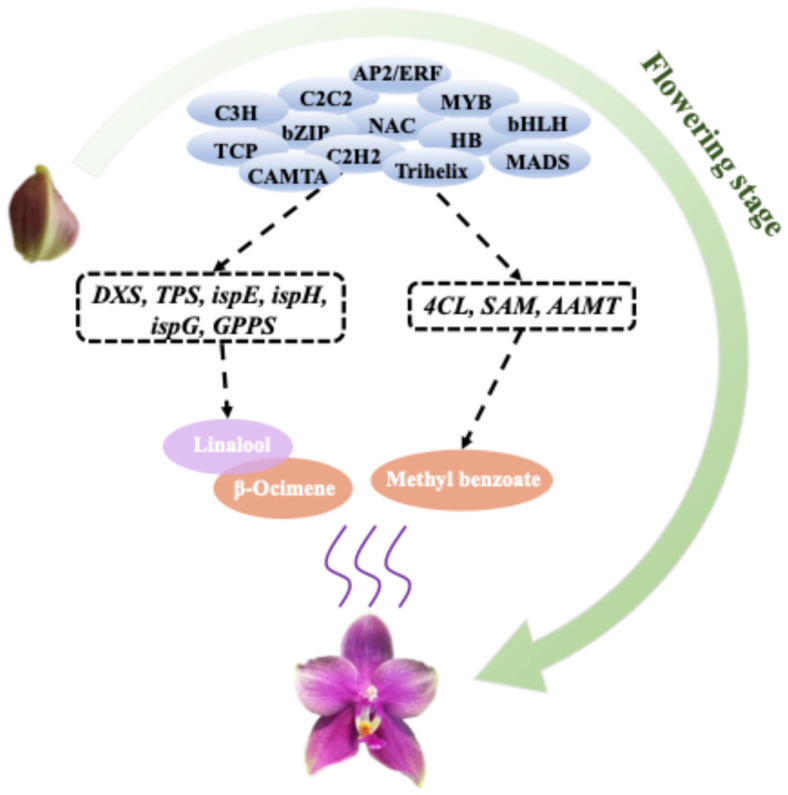
Schematic diagram of the mechanism of aroma release of *Phalaenopsis* Formosa Sweet Memory. The main aroma compounds of *Phalaenopsis* Formosa Sweet Memory were β-Ocimene, Linalool, and Methyl Benzoate. *DXS*, *TPS*, *ispE*, *ispH*, *ispG*, *GPPS 4CL*, *SAM*, and *AAMT* were key genes in the biosynthesis pathways of terpenoids and esters, which were co-expressed with aroma release. These structural genes were also regulated by transcription factors.

Based on integrated volatilomics and transcriptomics, this study elucidated the molecular mechanisms underlying aroma emission during the flowering process of *Phalaenopsis*. The main aroma compounds of *Phalaenopsis* Formosa Sweet Memory are Linalool, β-Ocimene, and Methyl Benzoate, and the release of aroma is significantly upregulated during the flowering process. Based on the aroma release patterns of 10 fragrant *Phalaenopsis* cultivars, it further confirmed the significance of Linalool, β-Ocimene, and Methyl Benzoate in the aroma components of *Phalaenopsis*. During the flowering process of *Phalaenopsis*, the structural genes involved in the pathways related to terpenoids and esters were significantly correlated and were regulated by transcription factors. Meanwhile, the expression patterns of *PAXXG356500_TPS*, *PAXXG333030_4CL,* and *PAXXG061420_SAM* were consistent with the release patterns of terpenoids or esters in *Phalaenopsis*, which may be a key factor in regulating aroma formation (Figure 7).

## Data Availability

The datasets generated for this study can be found in the National Centre for Biotechnology Information (NCBI) Sequence Read Archive under accession numbers SRR35845424, SRR35845425, SRR35845426, and SRR35845427.

## Supplementary Materials

The following supporting information can be downloaded at https://www.mdpi.com/article/10.3390/plants14233682/s1, Supplementary Figure S1: Quality control analysis of 10 *Phalaenopsis* samples for volatile determination. (A) Quality control assessment of VOCs’ profiling using mixed samples. (B) Coefficient of variation (CV) analysis. (C) PCA on 1583 VOCs in 10 fragrant *Phalaenopsis* cultivars. Supplementary Figure S2: Quality control analysis of samples from different flowering stages of *Phalaenopsis* Formosa Sweet Memory for volatile determination. (A) Pearson’s Correlation Coefficient for correlation analysis among samples. (B) Coefficient of variation (CV) analysis. (C) Quality control assessment of VOCs profiling using mixed samples. Supplementary Figure S3: Seven K-means clusters show differential trends of differential metabolites during flowering stages. Supplementary Figure S4: Overview of DEGs in the biosynthesis pathways of terpenoids and esters in *Phalaenopsis* Formosa Sweet Memory. Supplementary Figure S5: The gene expression patterns of the black, blue, brown, green, green-yellow, magenta, pink, purple, red, tan, turquoise and yellow modules in WGCNA. The upper part shows the clustering heatmap of genes within this module, with red indicating high expression, and green indicating low expression. The lower part shows the expression patterns of module feature values in different samples. Supplementary Figure S6: Analysis of the function and correlation of DEGs in MEbrown and MEblue. (A) KEGG pathway enrichment of DEGs in the MEblue. (B) KEGG pathway enrichment of DEGs in the MEbrown. (C) Correlation heatmap between aroma-related transcription factors expression levels and key odorant contents. (D) Correlation heatmap between aroma-related structural gene expression levels and key odorant contentsç Table S1: The HS-SPME and GC-MS conditions. Table S2: Summary of all differentially expressed genes. Table S3: Primers used for real-time quantitative PCR.

## Abbreviations

VOCs, volatile organic compounds; MVA, mevalonic acid; MEP, 2-C-methyl-D-erythritol-4-phosphate; G3P, glyceraldehyde 3-phosphate; IPP, isopentenyl diphosphate; DMAPP, dimethylallyl diphosphate; GPPS, geranyl diphosphate synthase; TPS, terpene synthase; PAL, phenylalanine ammonia lyase; dbEST, EST database; LOX, lipoxygenase; DGK, diacylglycerol kinase; GC-MS/MS, Gas Chromatography–Tandem Mass Spectrometry; TFs, transcription factors; HS-SPME, headspace solid-phase microextraction; FPKM, fragments per kilobase of transcript per million fragments mapped; PCA, principal component analysis; DEGs, differentially expressed genes; WGCNA, Weighted Gene Co-Expression Network Analysis; SAM, S-adenosylmethionine; 4CL, 4-Coumaroyl-CoA Ligase.
