# Supplementary material for: Multi-Omics Analysis Unravels the Biosynthesis and Regulatory Mechanisms of Floral Scent Across Various Cultivars and Developmental Stages in Phalaenopsis
Source: Plants (Basel). 2025 Dec 3;14(23):3682. doi: 10.3390/plants14233682 (PMC12694178; doi:10.3390/plants14233682)
Supplement: Supplementary file 1 [file plants-14-03682-s001.zip › Supplementary File/Figure S3.pdf]

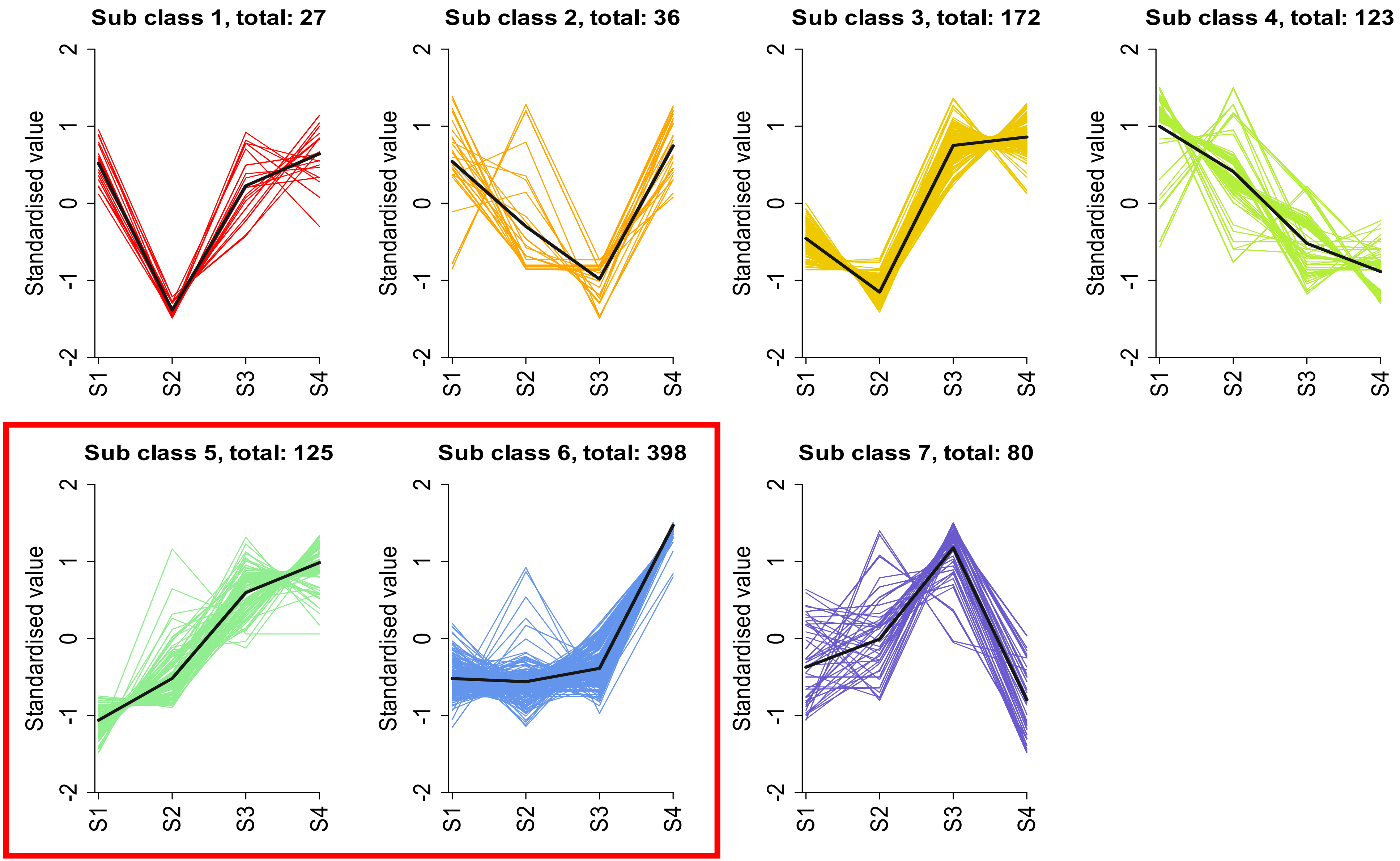
Sub class 1, total: 27
Sub class 2, total: 36
Sub class 3, total: 172
Sub class 4, total: 123
Sub class 5, total: 125
Sub class 6, total: 398
Sub class 7, total: 80
Standardised value
2
1
0
-1
-2
S1
S2
S3
S4
