# Supplementary material for: Multi-Omics Analysis Unravels the Biosynthesis and Regulatory Mechanisms of Floral Scent Across Various Cultivars and Developmental Stages in Phalaenopsis
Source: Plants (Basel). 2025 Dec 3;14(23):3682. doi: 10.3390/plants14233682 (PMC12694178; doi:10.3390/plants14233682)
Supplement: Supplementary file 1 [file plants-14-03682-s001.zip › Supplementary File/Figure S4.pdf]

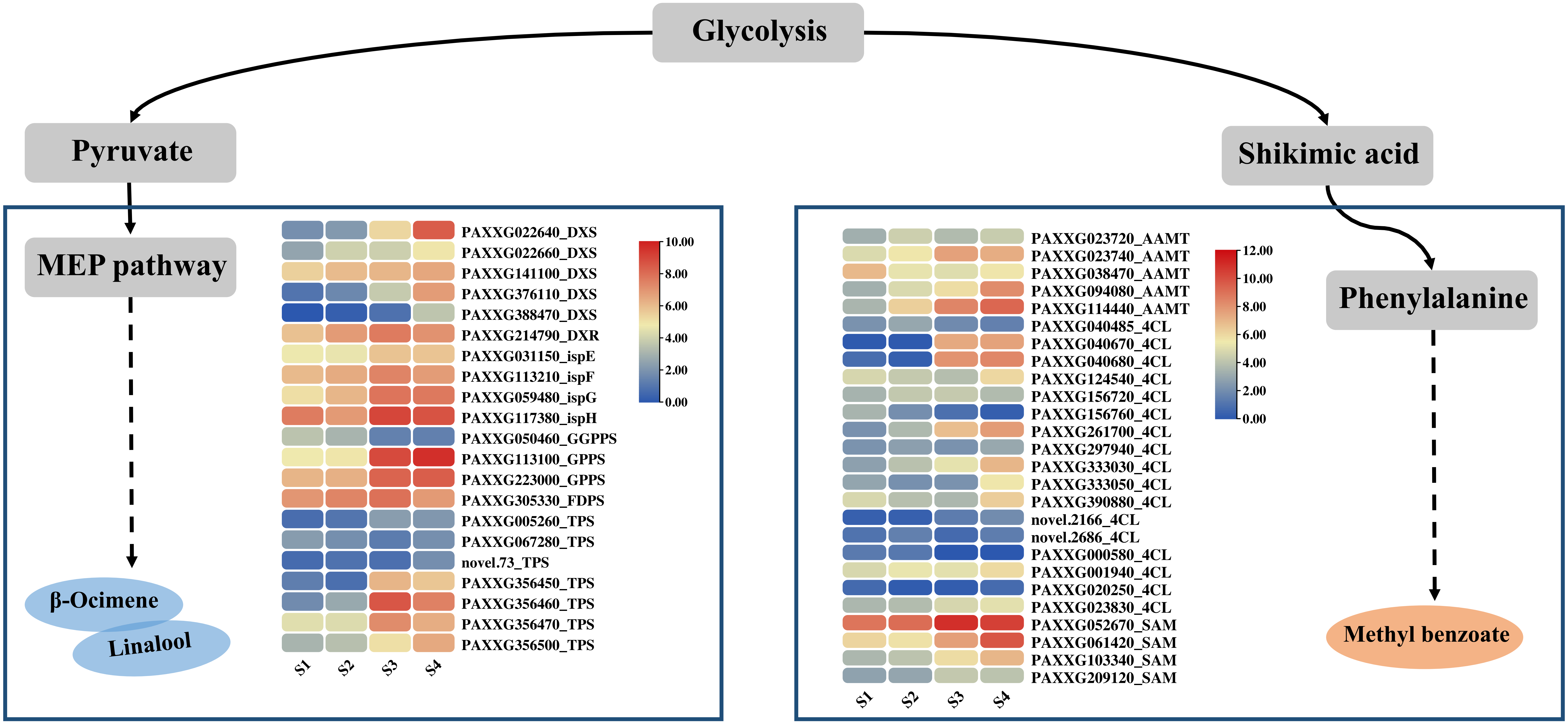

Glycolysis
Pyruvate
Shikimic acid
MEP pathway
PAXXG022640_DXS
PAXXG022660_DXS
PAXXG141100_DXS
PAXXG376110_DXS
PAXXG388470_DXS
PAXXG214790_DXR
PAXXG031150_ispE
PAXXG113210_ispF
PAXXG059480_ispG
PAXXG117380_ispH
PAXXG050460_GGPPS
PAXXG113100_GPPS
PAXXG223000_GPPS
PAXXG305330_FDPS
PAXXG005260_TPS
PAXXG067280_TPS
novel.73_TPS
PAXXG356450_TPS
PAXXG356460_TPS
PAXXG356470_TPS
PAXXG356500_TPS
S1
S2
S3
S4
10.00
8.00
6.00
4.00
2.00
0.00
β-Ocimene
Linalool
PAXXG023720_AAMT
PAXXG023740_AAMT
PAXXG038470_AAMT
PAXXG094080_AAMT
PAXXG114440_AAMT
PAXXG040485_4CL
PAXXG040670_4CL
PAXXG040680_4CL
PAXXG124540_4CL
PAXXG156720_4CL
PAXXG156760_4CL
PAXXG261700_4CL
PAXXG297940_4CL
PAXXG333030_4CL
PAXXG333050_4CL
PAXXG390880_4CL
novel.2166_4CL
novel.2686_4CL
PAXXG000580_4CL
PAXXG001940_4CL
PAXXG020250_4CL
PAXXG023830_4CL
PAXXG052670_SAM
PAXXG061420_SAM
PAXXG103340_SAM
PAXXG209120_SAM
S1
S2
S3
S4
12.00
10.00
8.00
6.00
4.00
2.00
0.00
Phenylalanine
Methyl benzoate
