# Supplementary figures and images for: Multi-Omics Analysis Unravels the Biosynthesis and Regulatory Mechanisms of Floral Scent Across Various Cultivars and Developmental Stages in Phalaenopsis
Source: Plants (Basel). 2025 Dec 3;14(23):3682. doi: 10.3390/plants14233682 (PMC12694178; doi:10.3390/plants14233682)

A

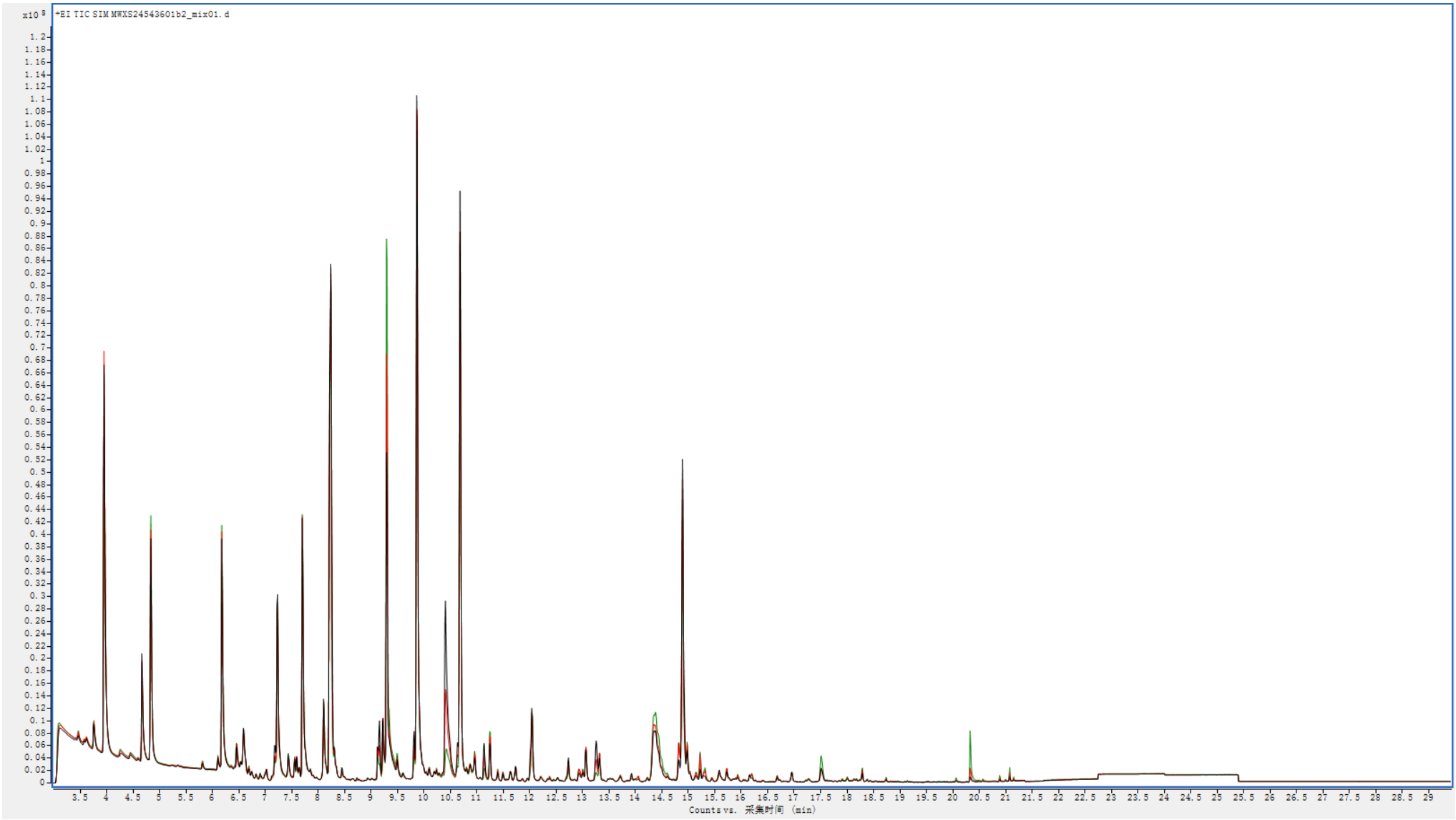

B

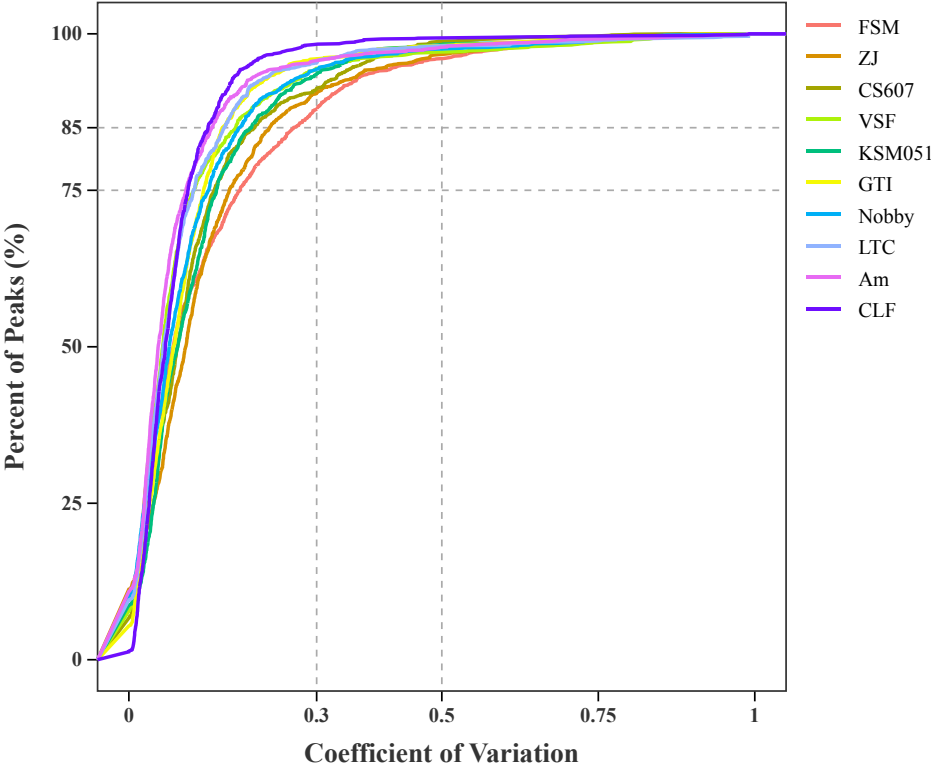

C

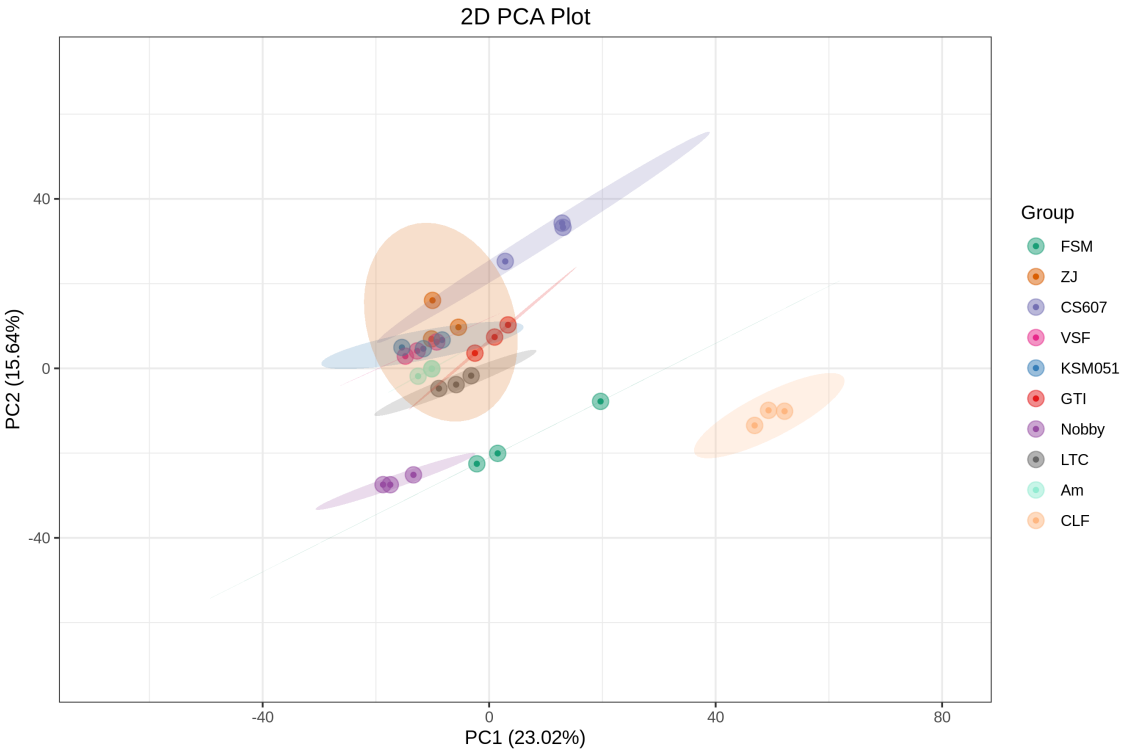

Supplement: Supplementary file 1 [file plants-14-03682-s001.zip › Supplementary File/Figure S1.pdf]

A

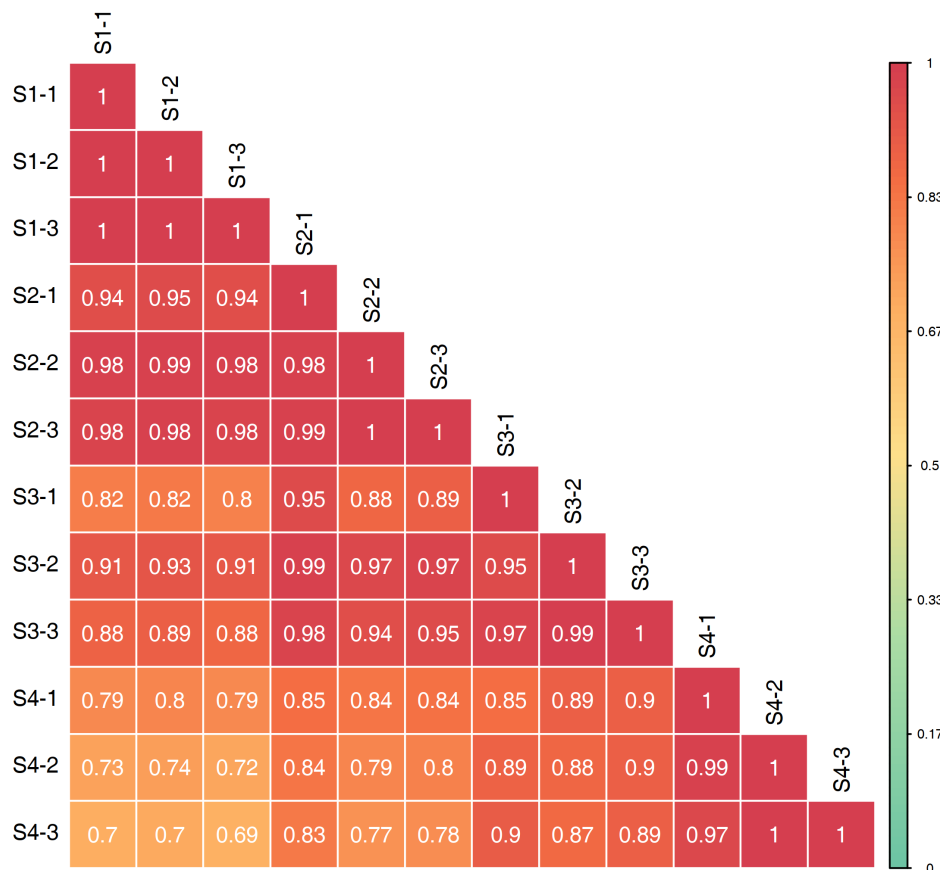

B

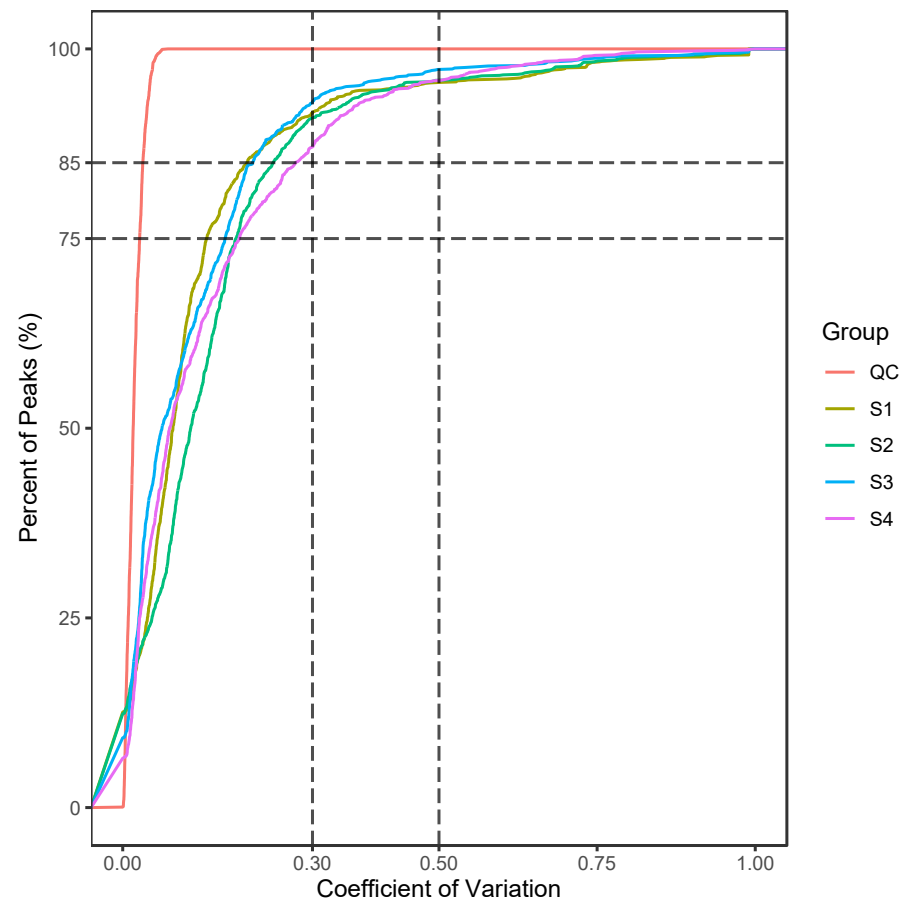

C

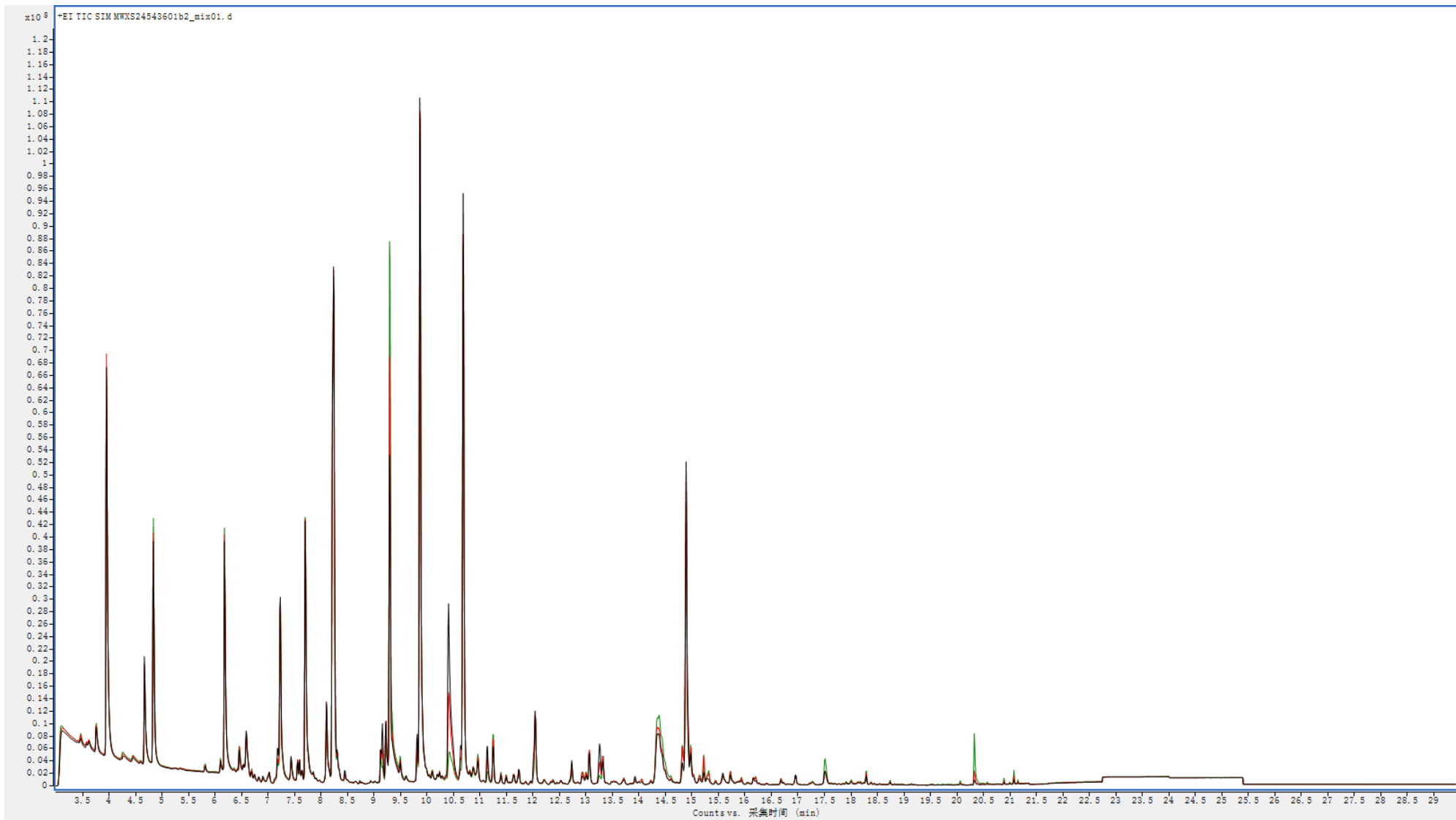

Supplement: Supplementary file 1 [file plants-14-03682-s001.zip › Supplementary File/Figure S2.pdf]

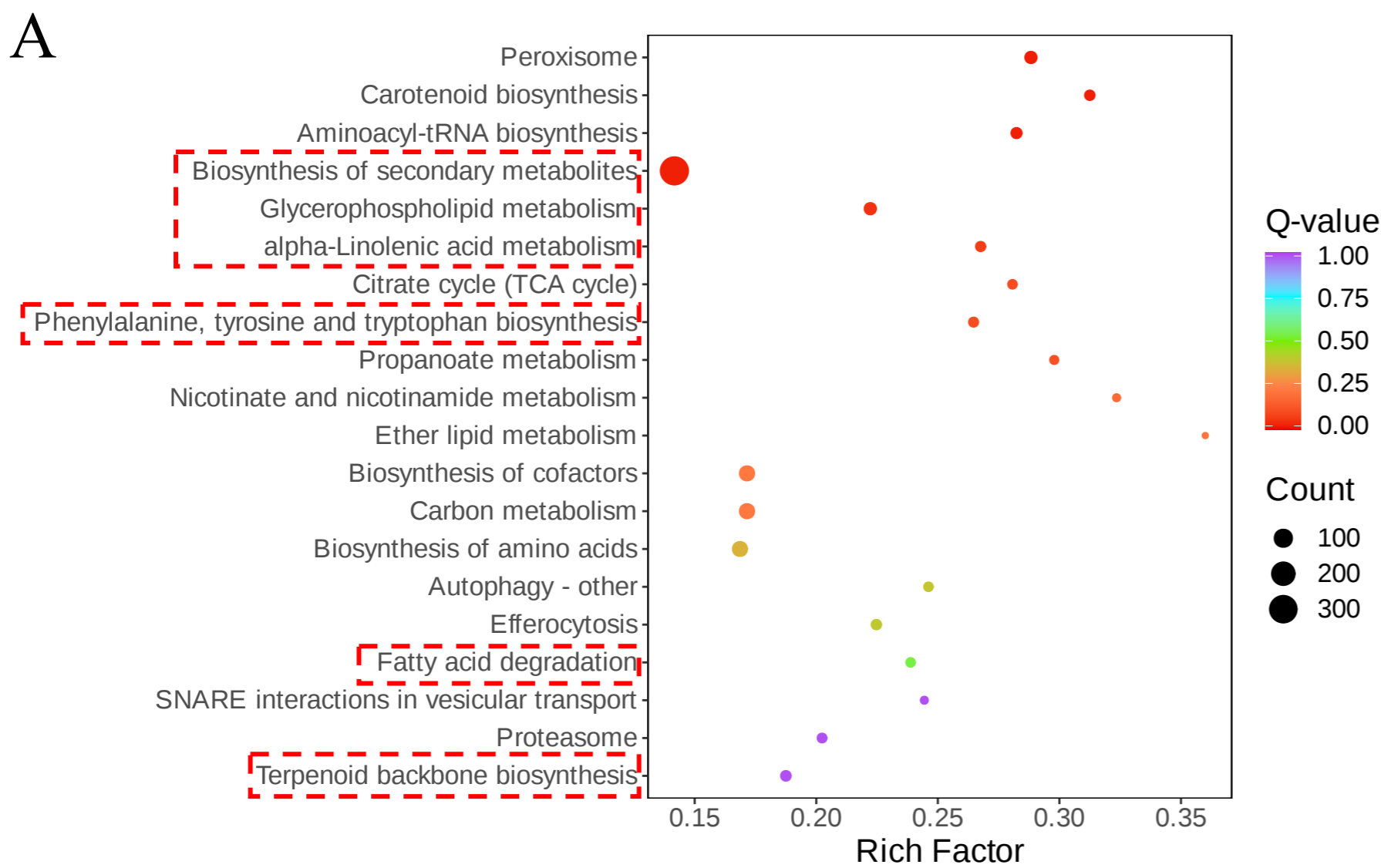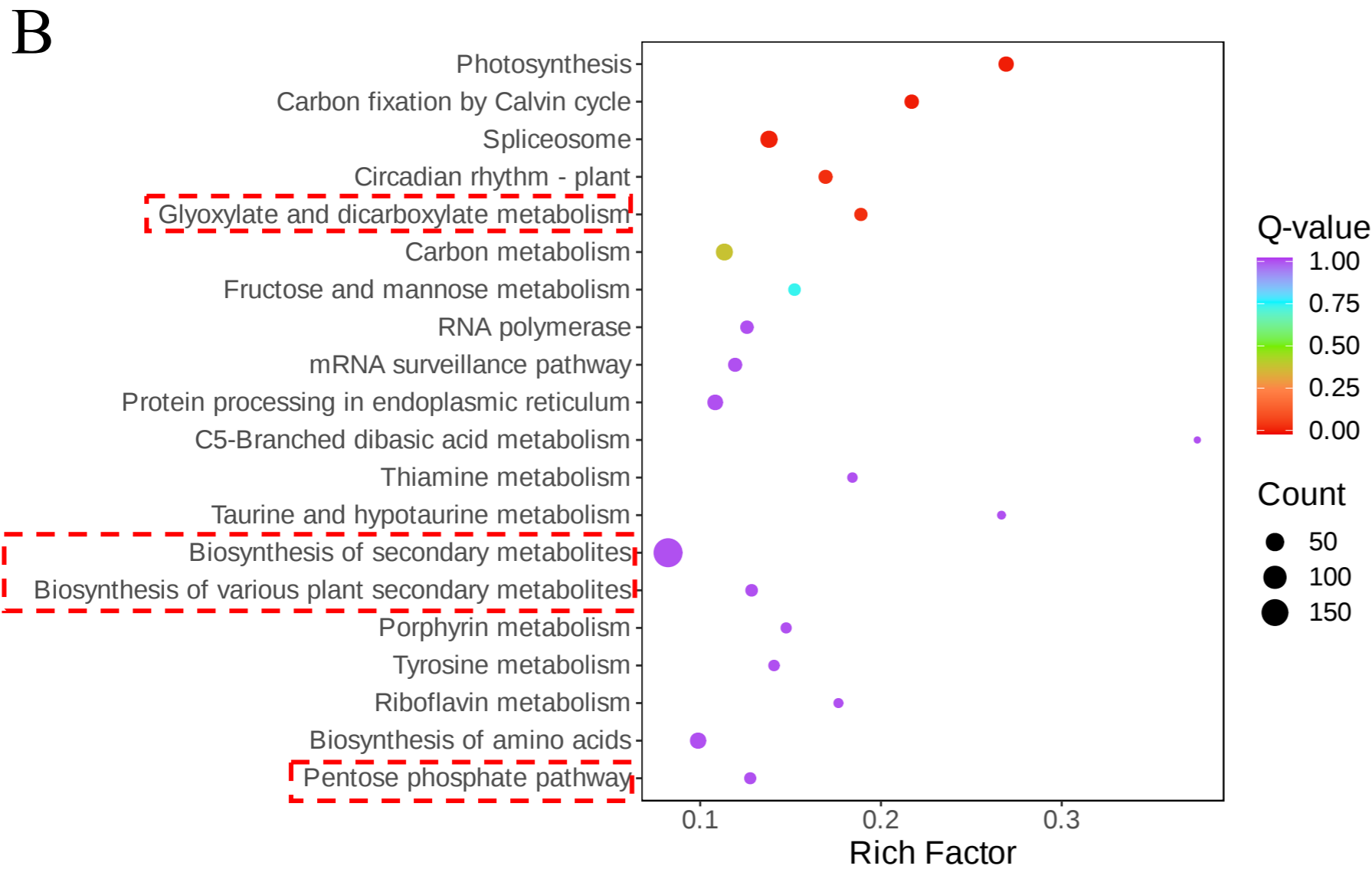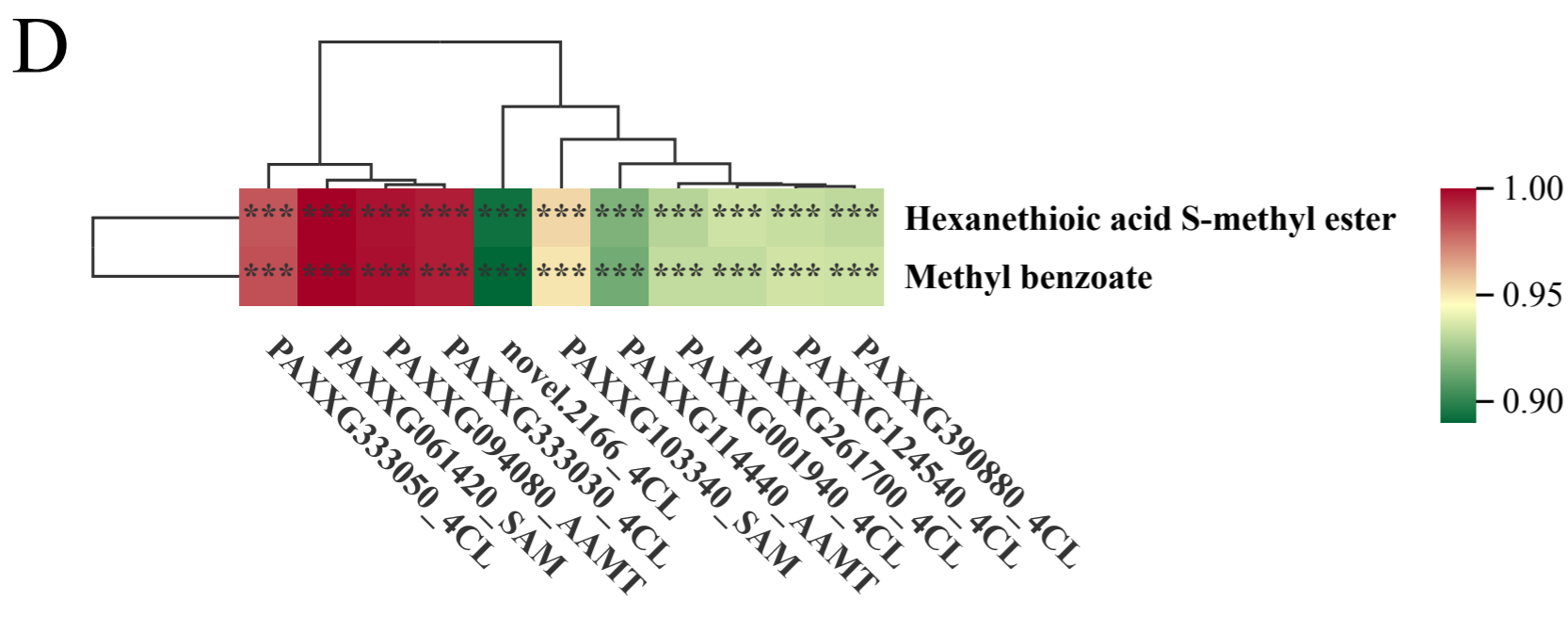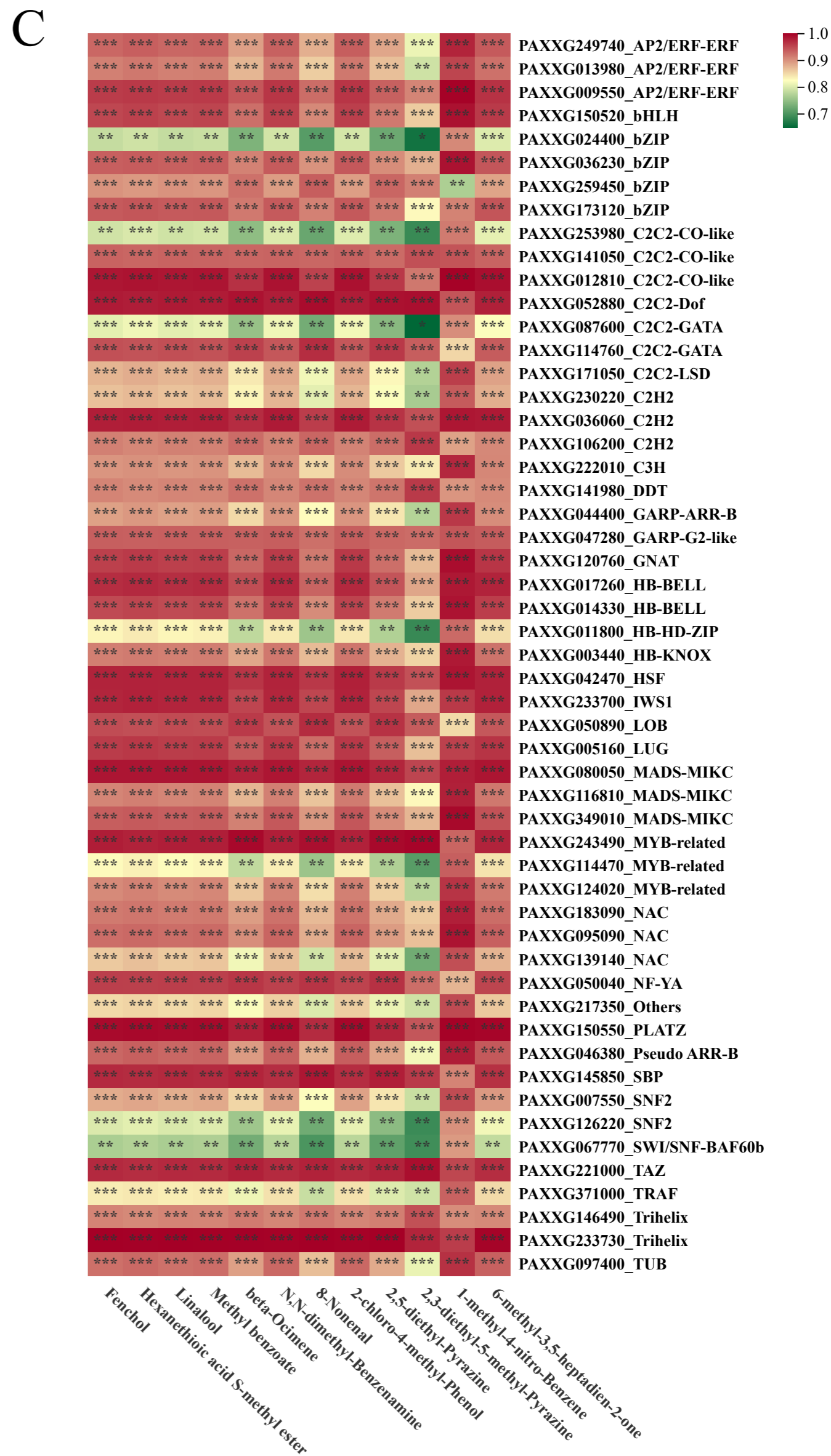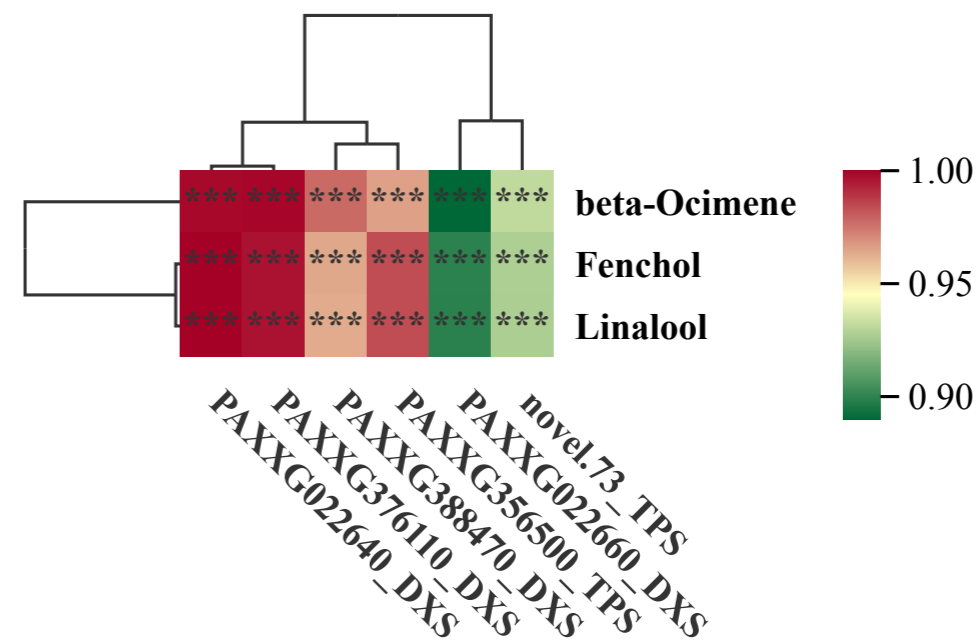

Supplement: Supplementary file 1 [file plants-14-03682-s001.zip › Supplementary File/Figure S6.pdf]

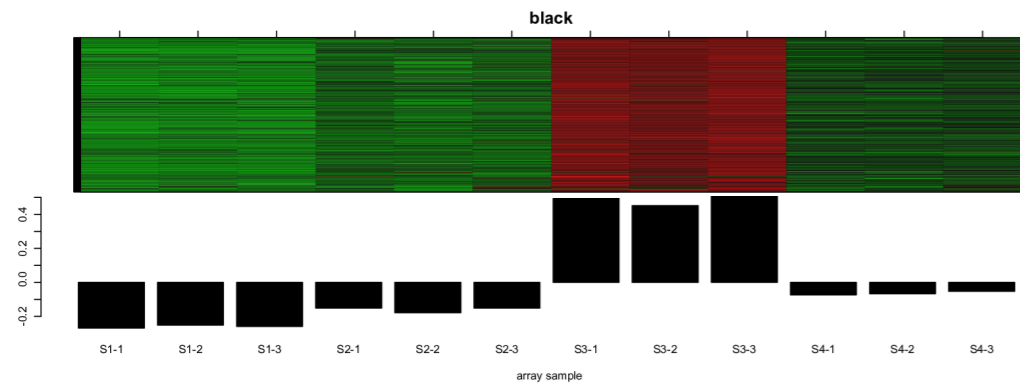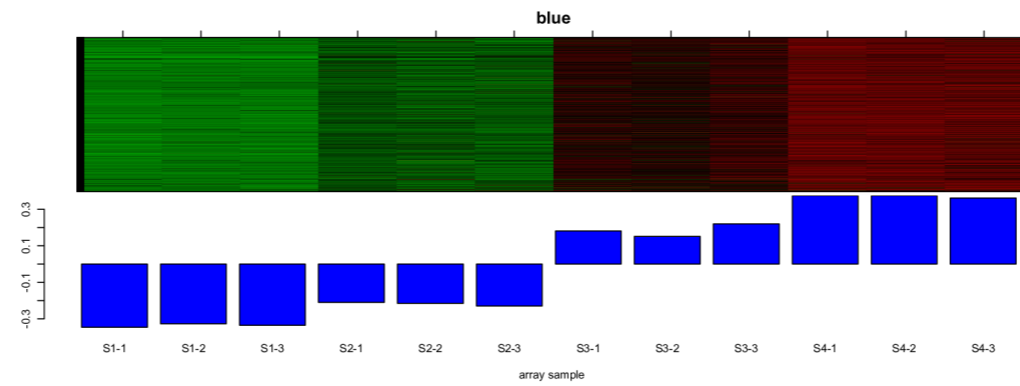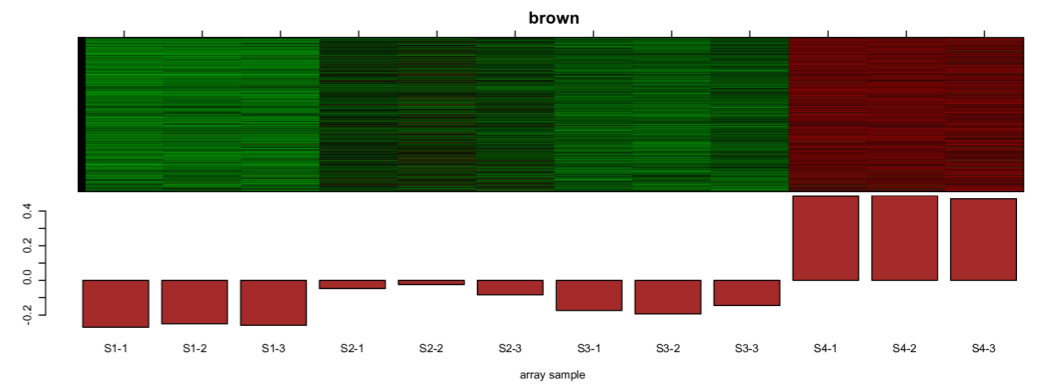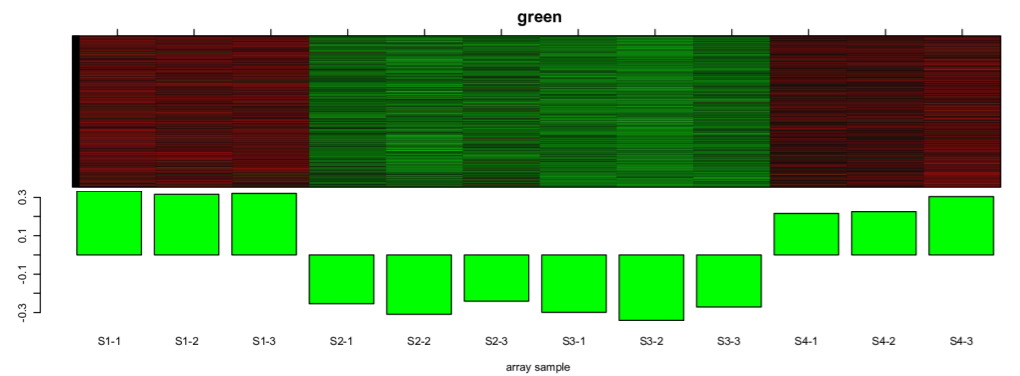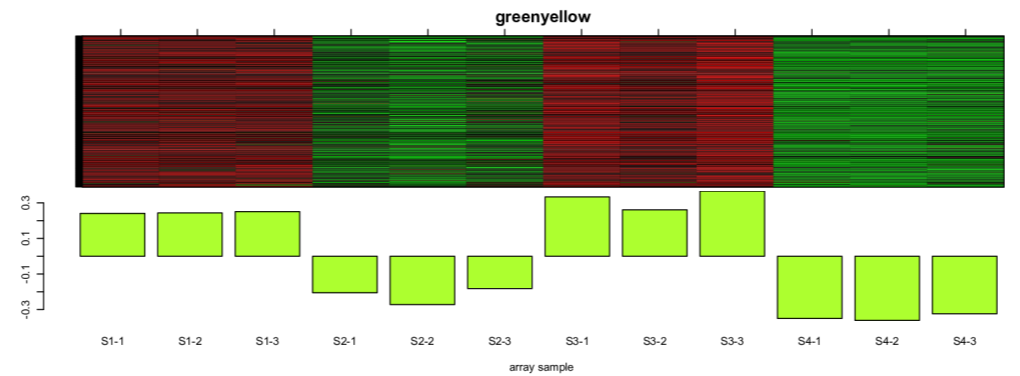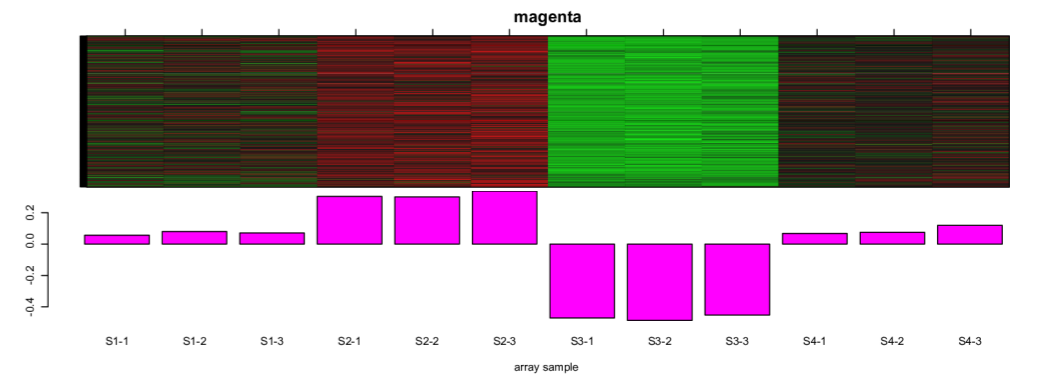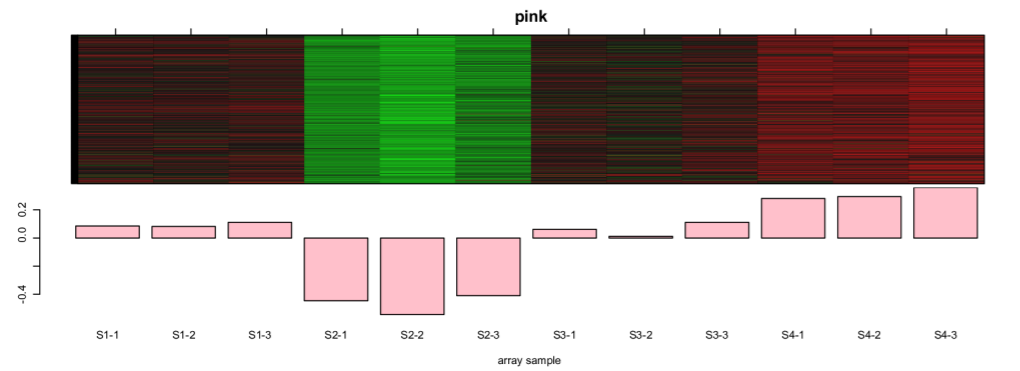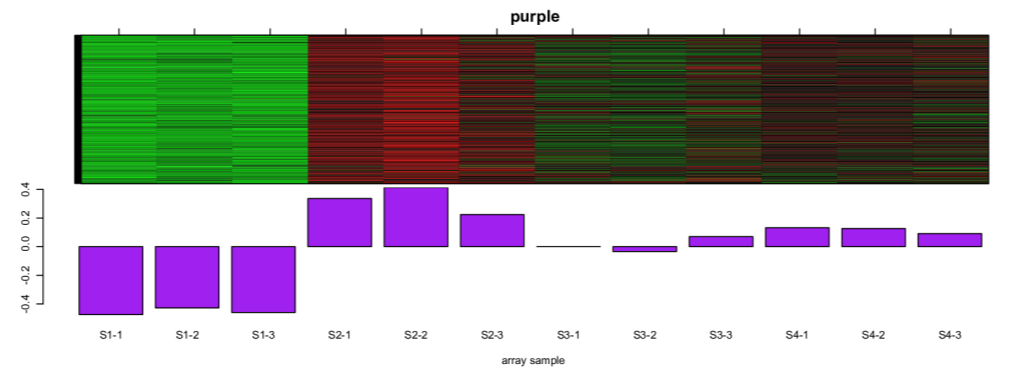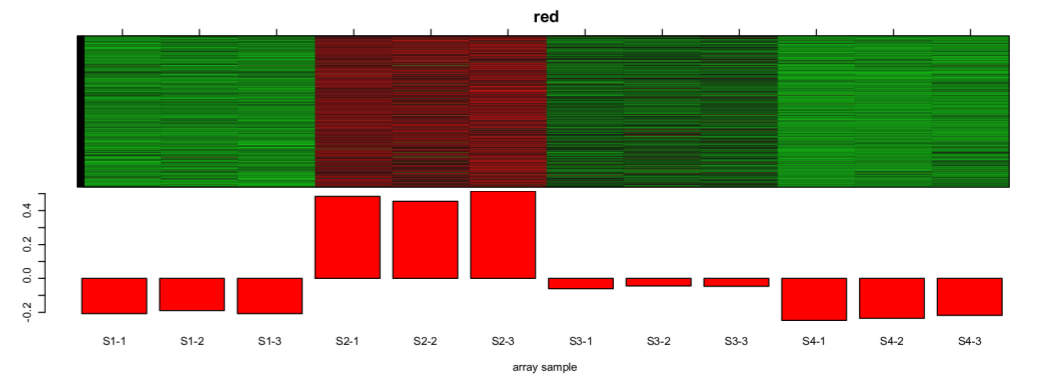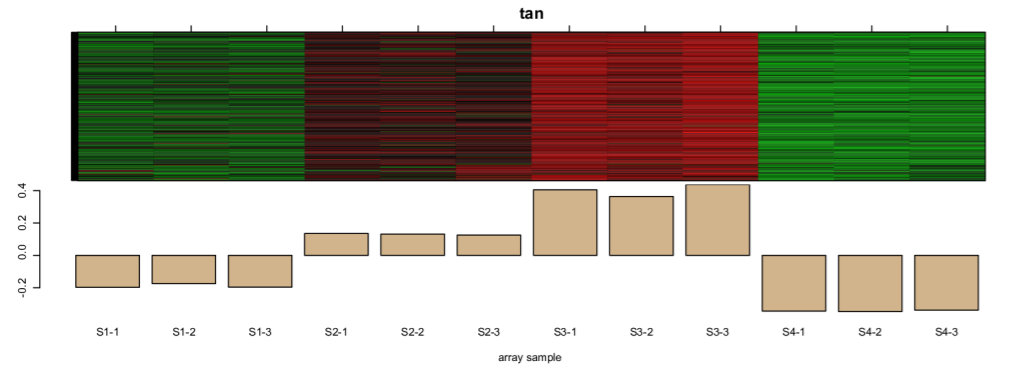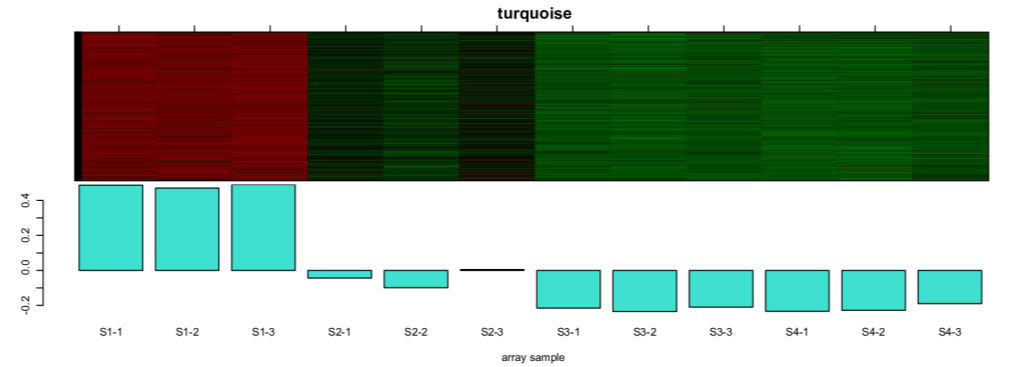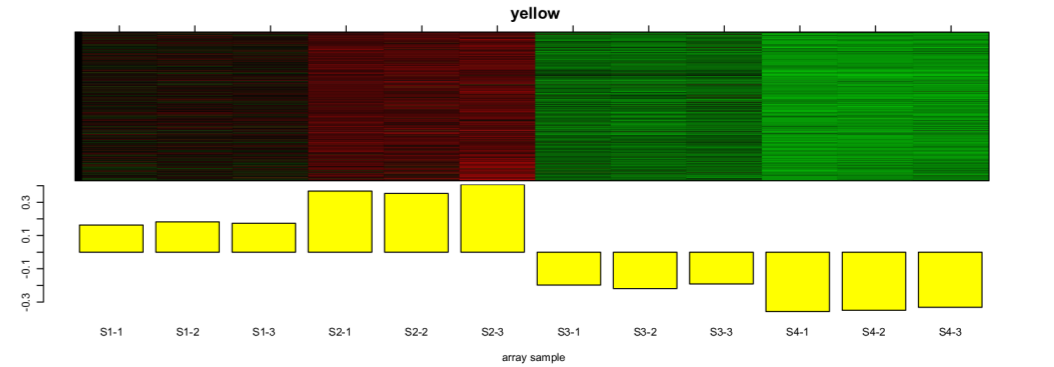

Supplement: Supplementary file 1 [file plants-14-03682-s001.zip › Supplementary File/Figure S5.pdf]
